# Microalgal co-cultivation -recent methods, trends in omic-studies, applications, and future challenges

**DOI:** 10.3389/fbioe.2023.1193424

**Published:** 2023-09-20

**Authors:** Raseena Naseema Rasheed, Asma Pourbakhtiar, Malihe Mehdizadeh Allaf, Maedeh Baharlooeian, Nahid Rafiei, Hossein Alishah Aratboni, Jose Ruben Morones-Ramirez, Flavia Vischi Winck

**Affiliations:** ^1^ Department of Botany, University of Kerala, Thiruvananthapuram, Kerala, India; ^2^ School of Chemical Engineering, College of Engineering, University of Tehran, Tehran, Iran; ^3^ Department of Civil and Environmental Engineering, Western University, London, ON, Canada; ^4^ Department of Marine Biology, Faculty of Marine Science and Oceanography, Khorramshahr University of Marine Science and Technology, Khorramshahr, Iran; ^5^ Regulatory Systems Biology Lab, Center for Nuclear Energy in Agriculture, University of São Paulo, Piracicaba, Brazil; ^6^ Centro de Investigación en Biotecnología y Nanotecnología, Facultad de Ciencias Químicas, Universidad Autónoma de Nuevo León, Parque de Investigación e Innovación Tecnológica, Apodaca, Nuevo León, Mexico; ^7^ Facultad de Ciencias Químicas, Universidad Autónoma de Nuevo León, Universidad Autonoma de Nuevo Leon (UANL), Av Universidad s/n, CD. Universitaria, San Nicolás de los Garza, Nuevo León, Mexico

**Keywords:** co-culturing systems, microalgae, bioprocess design, bioproduction, microbial consortia

## Abstract

The burgeoning human population has resulted in an augmented demand for raw materials and energy sources, which in turn has led to a deleterious environmental impact marked by elevated greenhouse gas (GHG) emissions, acidification of water bodies, and escalating global temperatures. Therefore, it is imperative that modern society develop sustainable technologies to avert future environmental degradation and generate alternative bioproduct-producing technologies. A promising approach to tackling this challenge involves utilizing natural microbial consortia or designing synthetic communities of microorganisms as a foundation to develop diverse and sustainable applications for bioproduct production, wastewater treatment, GHG emission reduction, energy crisis alleviation, and soil fertility enhancement. Microalgae, which are photosynthetic microorganisms that inhabit aquatic environments and exhibit a high capacity for CO_2_ fixation, are particularly appealing in this context. They can convert light energy and atmospheric CO_2_ or industrial flue gases into valuable biomass and organic chemicals, thereby contributing to GHG emission reduction. To date, most microalgae cultivation studies have focused on monoculture systems. However, maintaining a microalgae monoculture system can be challenging due to contamination by other microorganisms (e.g., yeasts, fungi, bacteria, and other microalgae species), which can lead to low productivity, culture collapse, and low-quality biomass. Co-culture systems, which produce robust microorganism consortia or communities, present a compelling strategy for addressing contamination problems. In recent years, research and development of innovative co-cultivation techniques have substantially increased. Nevertheless, many microalgae co-culturing technologies remain in the developmental phase and have yet to be scaled and commercialized. Accordingly, this review presents a thorough literature review of research conducted in the last few decades, exploring the advantages and disadvantages of microalgae co-cultivation systems that involve microalgae-bacteria, microalgae-fungi, and microalgae-microalgae/algae systems. The manuscript also addresses diverse uses of co-culture systems, and growing methods, and includes one of the most exciting research areas in co-culturing systems, which are omic studies that elucidate different interaction mechanisms among microbial communities. Finally, the manuscript discusses the economic viability, future challenges, and prospects of microalgal co-cultivation methods.

## Graphical Abstract

**Figure:**
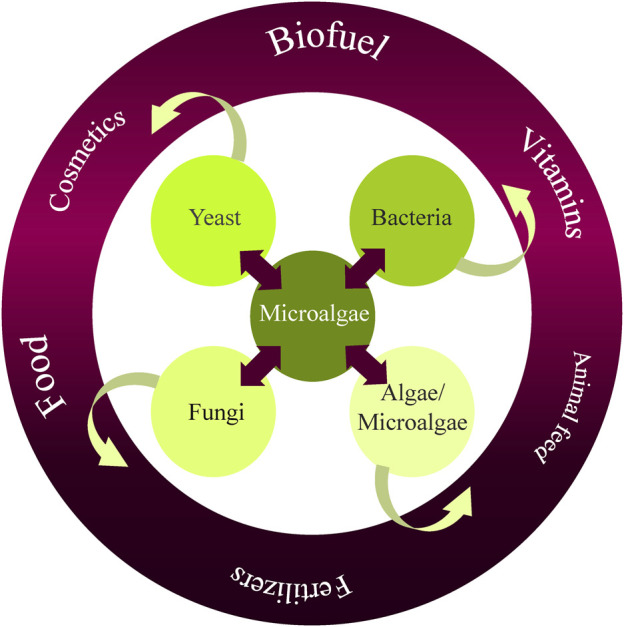

## 1 Introduction

The escalating human population’s increased demand for raw materials and energy sources is predicted to have a deleterious environmental impact characterized by elevated greenhouse gas (GHG) emissions. This trend is expected to continue in the near future (Barati et al., 2021a), given the ongoing process of industrialization, economic growth, and energy consumption (Masson-Delmotte et al., 2021). Consequently, to counterbalance these environmental threats and generate alternative bioproduct-producing technologies, sustainable methodologies are no longer an option but a necessity. One promising approach involves utilizing microbial consortiums or communities as a platform for developing diverse, sustainable applications that can outperform current wastewater treatment technologies, reduce GHG emissions, alleviate the energy crisis, and improve soil fertility (Das et al., 2021). The inclusion of microalgae species in such consortia addresses an essential aspect of circular economy and bioeconomy strategies: the generation of high-value compounds derived from the photosynthetic metabolism of oxygenic microalgae species.

Microalgae are photosynthetic microorganisms inhabiting marine and/or freshwater ecosystems. They exhibit a remarkably high CO_2_ fixation capacity compared to any other land plant, while also producing oxygen (Barati et al., 2021b). They can convert light energy into biomass and organic chemicals (Moreno-Garcia et al., 2017) and can consume atmospheric CO_2_ or industrial flue gases under specific circumstances, thereby reducing GHG emissions while producing biomass. Furthermore, microalgae can consume nutrients available in wastewater and collaborate with bioremediation (Barati et al., 2021b). Culturing domestic strains is typically straightforward, easy to maintain, and does not compete for arable lands (Lakshmikandan et al., 2020). Moreover, several species can exhibit an extraordinary capacity to adapt to different environmental niches, facilitating the bioprospecting of a microalgae species suitable for a particular environmental condition or its adaptation to a cultivation process (Lam and Lee, 2012). The potential for biotechnological and commercial applications of microalgae biomass is vast. It has been used in animal and human nutrition, cosmetics, biofertilization, the dyes industry, and antioxidant and pharmaceutical compounds (Rizwan et al., 2018). Additionally, bio-oil from microalgae can be used for biofuel production, in agricultural applications, controlling ammonia and balancing pH drops caused by nitrifying bacteria in an aquaponic system (Addy et al., 2017). They can also benefit plant growth in hydroponic systems by providing oxygen for the plant and utilizing the CO_2_ produced by respiration and exudation of crop roots for their growth, thereby inhibiting anaerobiosis in the crop’s root system (Huo et al., 2020).

**FIGURE 1:**
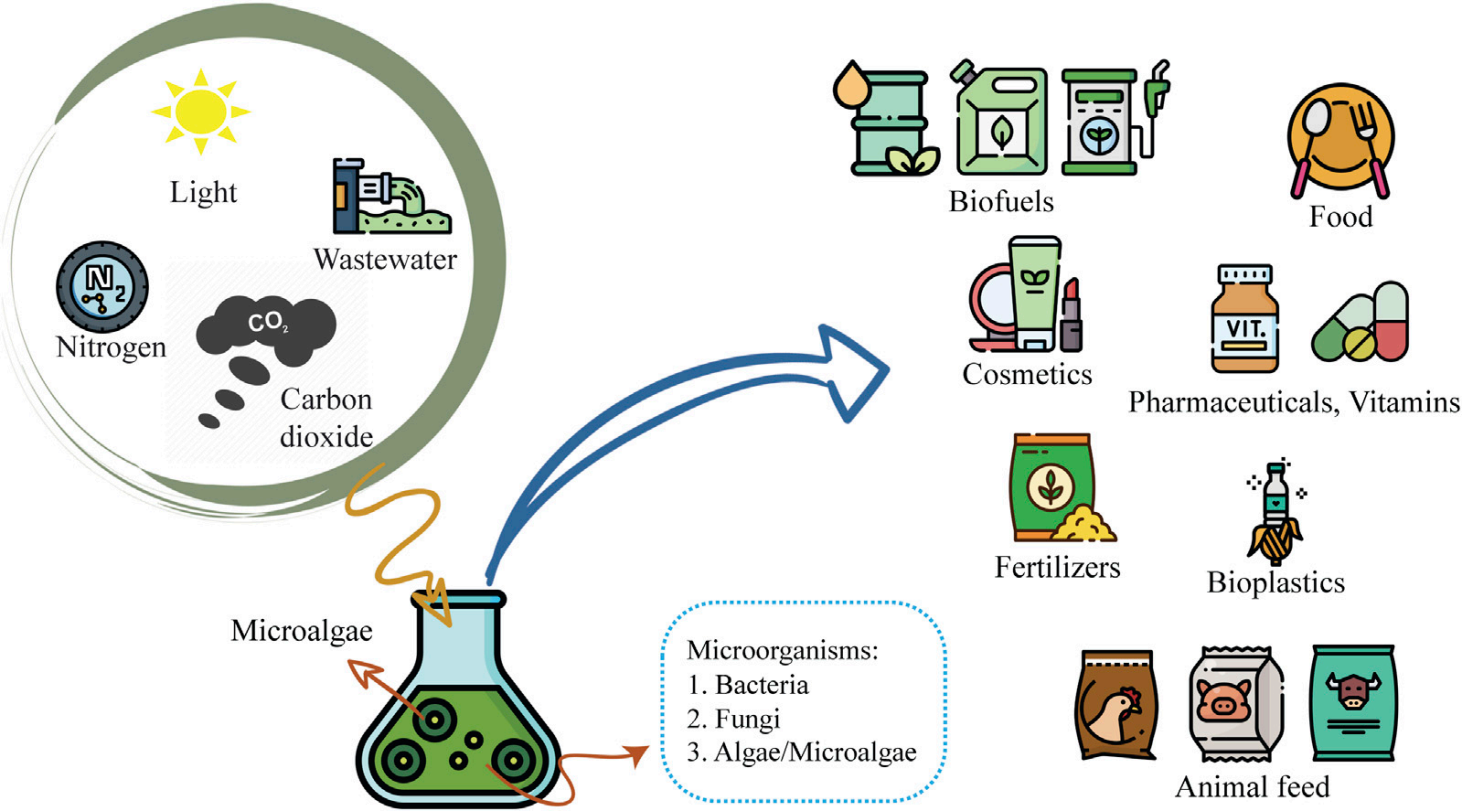
Advantages of microalgae co-culture systems and their potential applications.

Most microalgae cultivation studies have been focused on monoculture systems. However, monoculture open cultivation systems pose significant challenges due to contamination by other microorganisms, such as yeasts, fungi, bacteria, and other microalgae species. These instances of contamination can lead to low productivity, culture collapse, low-quality biomass, and nutrient loss. Accordingly, recent attention has been drawn to co-culture systems and the potential advantages of developing specific, robust microorganism consortia. Due to microalgae’s metabolic adaptability and capacity for survival in diverse environmental conditions, co-culturing microalgae with other microorganisms may circumvent the constraints of monoculture in open systems (Rashid et al., 2019). Co-culturing microalgae in consortia, at both small and large scales, has been developed and is utilized in biomanufacturing, with proposed applications in the food, agronomic, pharmaceutical, nutraceutical, chemical, biofuel sectors, and other industries associated with bioremediation and nutrient recycling strategies (Figure 1).

This review presents a thorough examination of research conducted in the last few decades, exploring the advantages and disadvantages of microalgae co-cultivation systems, including microalgae-bacteria, microalgae-fungi, and microalgae-microalgae/algae systems. The manuscript also addresses diverse uses of co-culture systems and growing methodologies and includes one of the most exciting research areas in co-cultivation systems, specifically, omics analysis, capable of elucidating different interaction mechanisms among microbial communities. Finally, the manuscript discusses the economic viability, future challenges, and prospects of microalgal co-cultivation methods.

## 2 Microalgal co-cultivation systems

**FIGURE 2:**
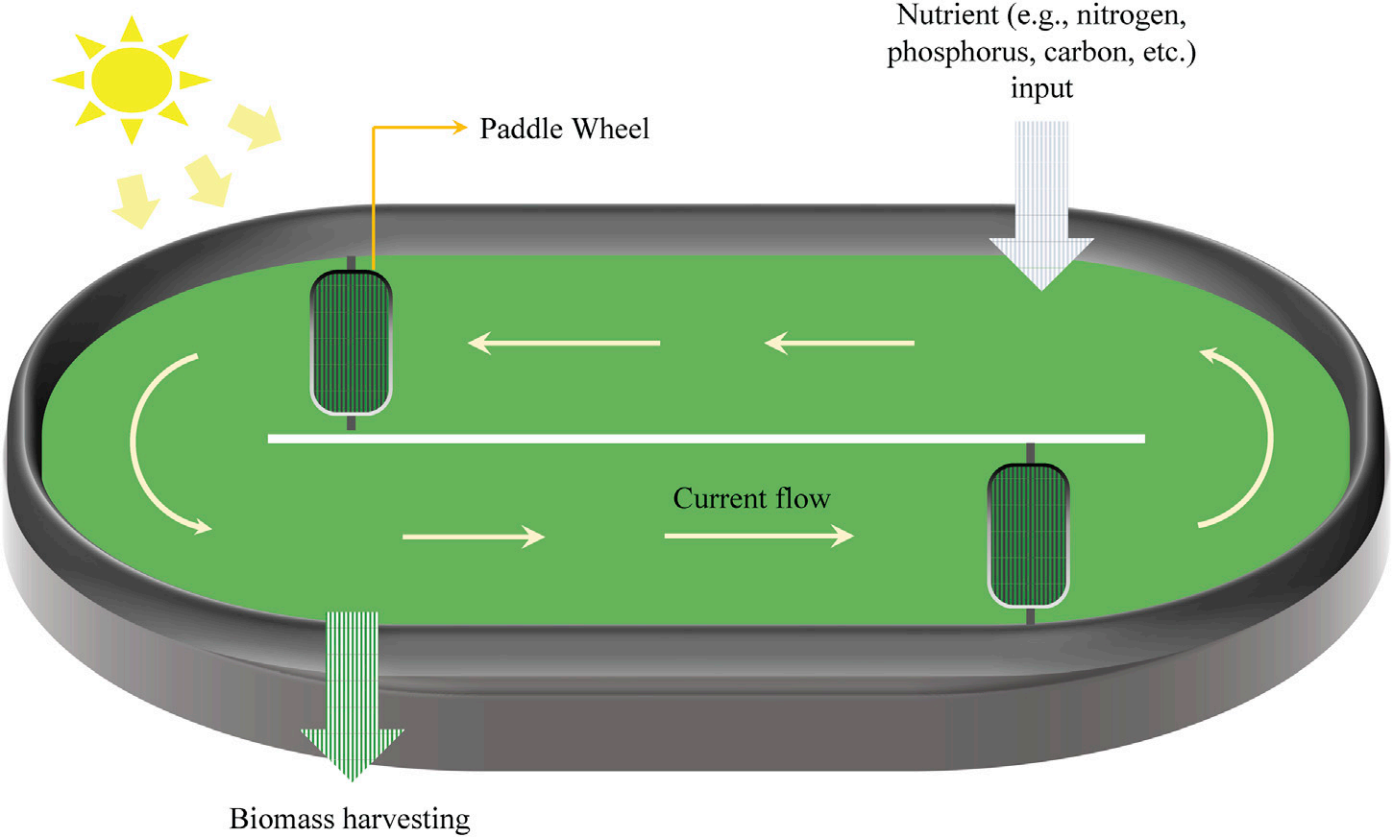
Schematic representation of the open pond system. The co-cultivation of microalgae in an open pond system is somewhat similar to the consortia in nature.

**FIGURE 3:**
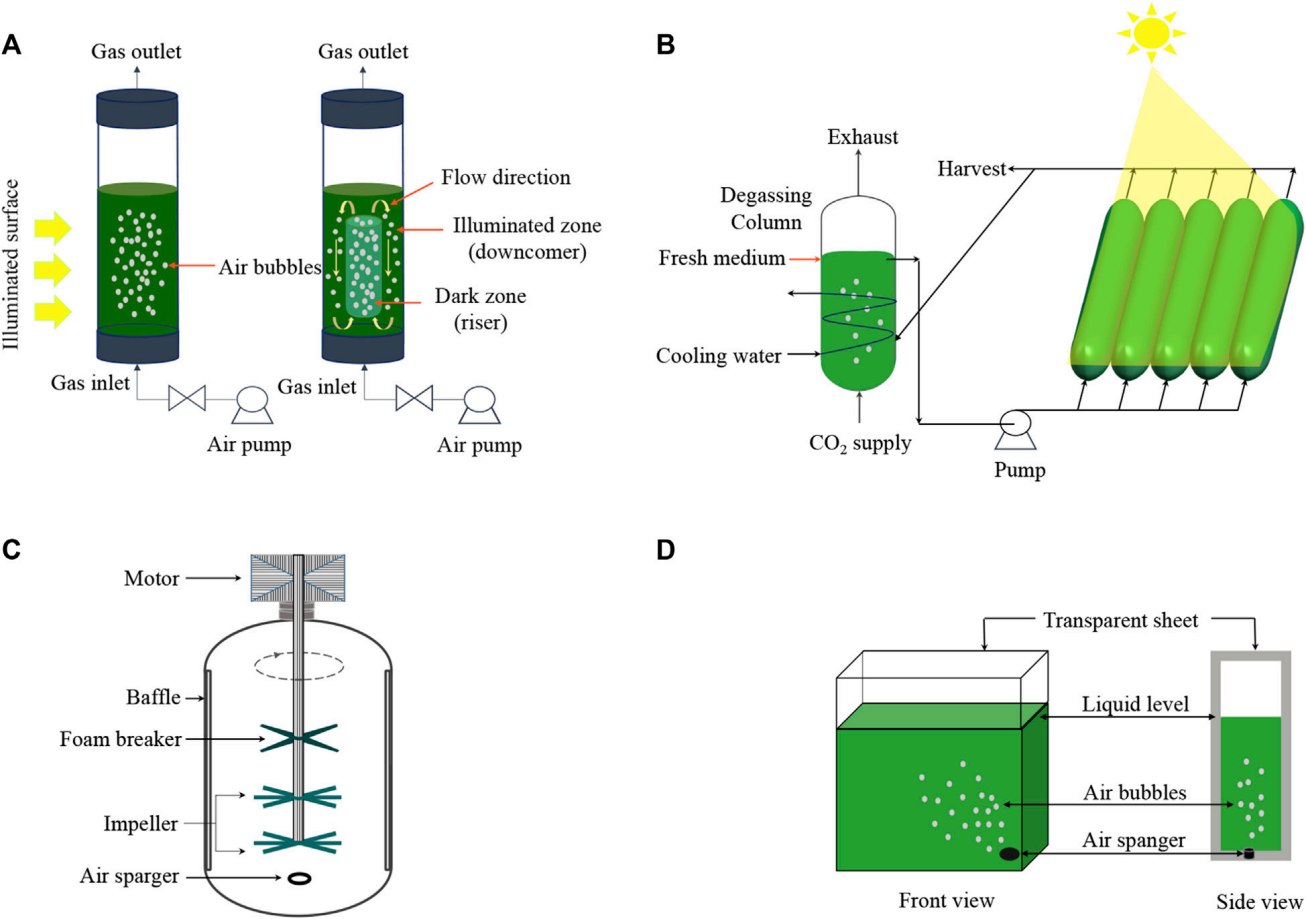
Types of photobioreactors for microalgae consortium. Vertical tube PBR **(A)**: Bubble column (left) and Airlift column (right); Horizontal tube PBR **(B)**; Stirred tank mechanism **(C)**; Flat panel PBR **(D)**.

Microalgae cultivation systems are a promising intersection of biology and sustainable industrial practice, offering potential for diverse product generation and bioremediation. These systems can be broadly classified into two categories: open and closed. An open system primarily comprises artificial ponds that are highly influenced by environmental fluctuations and are particularly susceptible to contamination by non-beneficial microorganisms (Figure 2). In contrast, closed systems consist of various photo-bioreactors (PBRs) (Figure 3), which require greater investments in initial infrastructure but are typically less vulnerable to cross-kingdom and cross-species contamination and can result in higher productivity of special high-value compounds.

Co-cultivation systems, an intersection of biology and sustainable industrial practice, hold promise for diverse product generation and bioremediation. The construction of these systems, whether open or closed, hinges on a clear understanding of the elements involved. Within these systems, we primarily encounter two types of cultures: axenic cultures, which host a single species, and non-axenic cultures, which are consortia of multiple microorganisms. The choice between these cultures depends on the goals of the co-culture, whether they be production, substrate consumption, or biomass accumulation. An essential consideration is whether survival of all constituent species is vital.

Designing these co-cultures involves applying principles of evolution: natural selection acting upon a diverse population (‘top-down’) or hand-picking strains (‘bottom-up’). Both approaches carry potential, dependent on the circumstances and the goals set. Biological interactions are as complex as they are vital. The range of possible interactions include from mutualism and commensalism to predation, parasitism, amensalism, and competition. A co-culture system may host several of these interactions simultaneously, even with just two species. Understanding these interactions is a prerequisite for effective system design.

Co-cultures come in various forms - suspended, flocculation, biofilms, and membranes, each with implications for reactor design and operational strategies. Another pivotal factor is illumination management. This is especially important for algae-based co-cultures, because light directly influences the productivity of photosynthetic microorganisms.

While creating minor adjustments to the co-culturing system can disrupt its balance, it is vital to establish new ways to design vessels, tanks, or reactors to anticipate culture behavior and facilitate future optimization. Identifying the composition of the extracellular chemical milieu, including metabolites, peptides, or proteins secreted by species within the consortium, is a significant step towards building consortium production (Lakshmikandan et al., 2021). However, we face challenges tracking molecular transfers between microbes and boosting the large-scale application of these technologies. One of the primary obstacles to further implementing microalgal biomass as an economically feasible feedstock is to achieve high biomass production coupled with the high yield of desired metabolites, cost-effective dewatering and harvesting of biomass, and a green and efficient procedure for product extraction (Kumar et al., 2020). Despite numerous attempts to increase microalgal productivity through nutritional, environmental, and physiological alteration-based cultivation, commercial success has been modest (Pierobon et al., 2018). Therefore, it is highly desirable to improve our understanding of the limitations and advantages of the current strategies of microalgae co-cultivation and the incorporation of recent knowledge generated through omics analysis into the perception and development of future co-cultivation systems towards increasing their applicability, especially in sustainable applications.

Next, we examine the primary methods currently employed for microalgae co-cultivation, highlighting the potential contributions of omics analysis data and recent knowledge acquired about microalgae metabolism. These findings may complement and enhance novel co-cultivation strategies.

## 3 Microalgae-bacteria co-cultivation

Establishing any type of association between microorganisms in the same cultivation system requires a deep understanding of their metabolic needs. The ideal situation for maintaining a biological consortium is where two or more species involved benefit from the interaction. This can be achieved in some cases where the products of one species’ metabolism can be metabolized by the other, and *vice versa*, to boost both growth and the desired biotechnological goal (e.g., bioremediation, chemical degradation, bioproduct synthesis, *etc.*).

Co-cultivation systems of microalgae and bacteria can be established for specific combinations of species where complex nutrient cycling patterns can be obtained, satisfying the needs of each organism through interactions with other members of the culture, resulting in either a symbiotic interaction or synergetic association, which are at least partially advantageous for both species in the microorganism community. The selection and optimization of this system for long-term sustainable applications require an understanding of the microorganism community and its structure, which is usually dynamic during processes that use waste or residue materials. This type of co-culture has been proposed for sustainable energy production, bioremediation (mainly for wastewater treatment), food, pharmaceutical, and medical industries (Padmaperuma et al., 2018; Yao et al., 2019). Currently, the application of microalgae-bacteria consortia has shown several advantages, such as bacteria stimulating the growth of microalgae by producing growth-promoting substances, vitamins, and cofactors (Dao et al., 2018), microalgae producing O_2_ through photosynthesis, which oxygenic bacteria can use, and in return, bacteria producing CO_2_ which can be fixed by microalgae photosynthetically (Makut et al., 2020), and microalgae secreting several complex molecules that may serve as a source of carbon and nitrogen for bacterial growth (Guo and Tong, 2014).

Microalgae species have the capability to fix atmospheric carbon dioxide through photosynthesis, which is then assimilated into organic compounds via the Calvin–Benson–Bassham (CBB) cycle, a crucial part of their primary carbon metabolism. In addition, the microalgae’s respiration produces oxygen, which can benefit heterotrophic bacteria. Additionally, microalgae can release dissolved organic matter (DOM), dissolved organic carbon (DOC), dissolved organic nitrogen (Cieplik et al., 2018), and dissolved organic phosphorous (DOP), which serve as nutrient sources for bacterial growth. Bacterial re-mineralization of these organic nutrients into inorganic forms can further promote microalgae growth (Amin et al., 2012; Borowitzka and Moheimani, 2013; Thompson and Zehr, 2013). Apart from nutrient cycling, microalgae and bacteria have a beneficial interaction via the exchange of molecules such as siderophores, which enhance iron supply to microalgae during growth (Vraspir and Butler, 2009). Microalgae and bacteria can also synthesize different vitamins, including B12, B1, and B7. This are essential for microalgal growth, establishing another instance of mutually beneficial interaction between the two microbial communities (Yong et al., 2014).

### 3.1 Microalgae-bacteria co-cultivation methods

There are two major types of interactions that can be seen in co-cultures: mutualism and commensalism. In a mutualistic relationship, both organisms gain benefits from the interaction. On the other hand, commensalism refers to a relationship where one organism benefits, and the other is unaffected. These relationships can be complex, with outcomes dependent on the specific organisms and environmental conditions involved. Both of these microalgae-bacteria interactions in co-cultivation have been widely used for various applications through several methods, including direct mixing, pelletization and flocculation, encapsulation, biofilm formation, cell droplets synthesis, membrane separation, dialysis tube system, and agar. Each of these methods has its own advantages and drawbacks, such as scalability, ease of implementation, and yield potential, which should be considered when selecting the appropriate method for a particular application. A summary of these methods is presented in Table 1.

**TABLE 1:** Microalgae-bacterial co-culture systems used for bioremediation, biomass, and lipid productions (T-temperature).

Microalgae/bacteria	Product/application	Interaction	Culture specifications	Yield	Reference
*C. reinhardtii/A. chroococcum*	Enhance biomass and lipid production	Mutualism	Flask, T- 28 ^O^C, Time- 48 h	Lipid productivity 141.86 mg/(L·day), 19.4 times	Xu et al. (2018)
*C. variablis/I. loihiensis*	Increased biomass, protein, lipid yield	Mutualism	Flask, the spectral range of 400–700 nm, light- 60–120 μmol photons m^−2^ s^−1^	20%, 19.70%, 30% increase in biomass, lipid, protein respectively	Rajapitamahuni et al. (2019)
*Characium* sp.*/P. composti*	Biomass yield and lipid	Mutualism	Erlenmeyer Flask, T- 25°C, light- 50 μmol photons m^−2^ s^−1^, Time- 20days	69% biomass yield	Berthold et al. (2019)
*C. vulgaris/B. licheniformis*	Bioremediation of phosphorous	Mutualism	Flask, T- 28 ± 1 ^O^C, light/dark ratio = 12 h/12 h, light- 120 μmol photons m^−2^ s^−1^	Phosphorus removal rate reached 82.21%, which was 19.10% higher than the control	Luo et al. (2021)
*C. vulgaris/A. brasilense*	Bioremediation of ammonium, phosphorous	Commensalism	Chemostat bioreactor, T- 28 ± 2°C, Time-6 days, light- 30 μmol photons m^−2^ s^−1^	100% NH_4_^+^ removal after the fourth cycle, and 83% PO_4_, removal after the first cycle	De-Bashan et al. (2002)
*C. vulgaris/B. licheniformis*	Bioremediation of nitrogen, phosphorous	Mutualism	Flasks, T- 25°C, pH 7, light- 30 μmol photons m^−2^ s^−1^	86% NH_4_^+^ removal	Liang et al. (2013)
*B. braunii/Rhizobium* sp	Hydrocarbons for biofuels	Mutualism	Cylindrical photobioreactor, T-25 ± 2°C, light- 24 h dark cycle, light- 30 μmol photons m^−2^ s^−1^	Optimize biomass production by enhancing growth	Rivas et al. (2010)
*C. sorokiniana/A. brasiliense*	Starch production	Mutualism	Flasks, T- 27 ± 2 ^O^C, Time- 96 h	Enhanced starch production (23.15 times)	Lutzu and Dunford (2018)
*C. sorokiniana/Chlorella* sp.*/K*. *pneumonia* and *A. calcoaceticus*	Enhanced production of diesel oil, jet fuel, and fuel for stoves, biomass yield, bioremediation	Mutualism	Automated photobioreactor, T- 30°C, light: dark cycle- 16:8 h, light- 250 μmol photons m^−2^ s^−1^	3.17 g L^−1^, 99.95% and 95.16% biomass concentration, total nitrogen, and COD removal efficiency respectively	Goswami et al. (2019)
*T*. *weissflogii/B. infantis*	increased biomass growth and lipid accumulation	Mutualism	Flask, T- 24^O^C, salinity- 30 ppt, light:dark cycle- 12:12 h, light intensity-1,200 Lux, aeration- 24 h	1.13 ± 0.03 mg/(L/day) increase in lipid productivity	Yee et al. (2021)
*C. vulgaris/P*. *putida*	Removing nitrogen, and organic carbon	Mutualism	Flasks, T- 25^O^C	80% of nitrogen reduction (40 mg N/L)	Mujtaba et al. (2015)
*C. vulgaris/S. rosealbus*	Biomass and lipid productivity	Mutualism	Flasks, T- 27 ± 1^O^C,light- 100 μmol photons m^-2^ s-^1^)	Biomass (29%) and lipid productivity (57%)	Lakshmikandan et al. (2021)

#### 3.1.1 Direct mixing

Direct mixing is a commonly used method for establishing a consortium between microalgae and bacteria. This method involves the physical contact between the two organisms, leading to the exchange of signaling molecules and metabolites, as well as the competition for nutrients in an uncontrolled manner. Direct mixing is often employed for the cultivation of microorganisms for bioremediation, hydrogen, and biofuel production, and is known to lead to better outcomes in terms of bioactive compound production when compared to monocultures (Tran et al., 2010). An example of its potential can be seen in a study that observed a mutualistic relationship between the dinoflagellate *Prorocentrum minimum* and the bacterium *Dinoroseobacter shibae*. The study showed that direct mixing can lead to a symbiotic relationship where both organisms equally benefit (Wang et al., 2014). However, generalizations from these findings should be taken with caution, as every co-culturing situation has its unique characteristics.

#### 3.1.2. Pelletization and flocculation

Establishing specific consortia of microorganisms can be achieved by exploiting the flocculating properties of one or both partner cells, which can be induced by compounds released by one member of the consortium. This process results in the formation of pellets or aggregates of cells, which can optimize symbiosis and improve settling ability, especially of microalgae species (Powell and Hill, 2013). For example, a pH-dependent and reversible aggregation process was observed in *Bacillus* sp. with *Nannochloropsis oceanica*, which retained its ability to aggregate after fixation with paraformaldehyde, allowing for cell reuse (Powell and Hill, 2013). Co-cultivation of *Chlorella vulgaris* with different species of bacteria has been shown to cause microalgal flocculation, resulting in improved biomass yield and metabolite production (Lee et al., 2013). Similarly, consortia between *Halomonas* sp. and *Micrococcus* sp.*, Staphylococcus* sp.*,* or *Pseudomonas* sp. have also been reported to result in microalgal flocculation (Okaiyeto et al., 2013).

#### 3.1.3 Biofilms

Biofilm-based co-cultivation of microalgae and bacteria has gained recent attention due to its potential for high efficiency in collecting and dewatering cell suspensions. It is considered an alternative method for producing biomass that overcomes the drawbacks of suspended cultivation systems, while meeting the requirements for generating biomass for biofuel coupled with wastewater treatment (Kesaano and Sims, 2014; Berner et al., 2015). Most microalgal biofilms are composed of bacteria and microalgae, which are typically inexpensive, accessible, and reusable. Biofilm-based cultivation provides the microalgae with more exposure to light than when suspended in a liquid medium (Zhang et al., 2018), which improves biomass productivity, shortens hydraulic retention times (Boelee et al., 2014), makes it easier to control the cell growth area, and appears to have high efficacy in wastewater treatment (Ma et al., 2018). The improved biomass and pigment synthesis of aquatic photosynthetic microalgae biofilms were reported in the presence of *Bacillus stratosphericus* (Miranda et al., 2017). Despite these potential advantages, the complexities of biofilm interactions and management must not be overlooked.

#### 3.1.4 Encapsulation

In this method, one microorganism is immobilized in beads and co-cultured with the other microorganisms in a liquid medium, increasing the chances of biomass reuse. Co-culturing of *Synechococcus* sp. (cyanobacterium) beads with *Chlamydomonas reinhardtii* (microalgae) improved the growth and lipids production of the microalgae and increased the possibility of recycling the beads (Magdouli et al., 2016). Co-immobilization and co-encapsulation of microalgae and bacteria in alginate beads prevent the ingress of external microbiota and the release of immobilized microorganisms into wastewater, making them suitable for bioremediation processes to reduce ammonium and phosphorous from wastewater. However, the growth suppression is limited by the native wastewater bacterial community (Covarrubias et al., 2012). The microalgae *Haematococcus pluvialis* is widely studied in encapsulation systems for food applications. Cyst cells of *H. pluvialis* generated under unfavorable environmental conditions contain a substantial amount of astaxanthin, a powerful antioxidant. Due to its sensitivity to light, oxygen, and high temperatures, astaxanthin extraction has been studied using encapsulation techniques (Sarada et al., 2006; Zhao T. et al., 2019).

#### 3.1.5 Cell droplets

The cell droplets technique, although not commonly used in reactor settings, has gained attention for its ability to facilitate the culturing of microorganisms that are difficult to cultivate under laboratory conditions. This method involves developing aqueous two-phase systems with polymers to create water-in-water emulsion systems that promote multi-organism aggregation, allowing individual cells to represent single batches of cultivation. Symbiotic microorganisms have been successfully isolated using this technique. In a previous report, an aqueous two-phase system was used to trap bacterial colonies within magnetic dextran phases and suspend them as cell droplets to algal colonies in a polyethylene glycol phase, resulting in improved communication between the droplet colonies (Byun et al., 2013). This method has been described as high-throughput screening of cell-to-cell interactions (HiSCI) in algae growth-supporting bacteria.

#### 3.1.6 Membrane separation (vessel chambers)

The membrane separation technique involves culturing microorganisms in separate compartments of a vessel, connected by a semipermeable membrane that allows the diffusion of metabolites between the chambers. This method has been used to study ecological systems such as predator-prey and phytoplankton communities (Paul et al., 2013). For example, the co-culture of *D*. *shibae* and *Thalassiosira pseudonana* separated by a membrane resulted in high metabolite diffusion rates, with the bacterium products influencing the metabolic profile of *T. pseudonana* cells and enhancing their amino acid content (Paul et al., 2013). However, the success of this method is dependent on the nature of the exchanged molecules and the positive or negative effects of allelopathic interactions (impacts induced by the products released in the medium) on cell growth. The interaction of *Oocystis marsonii* with *Microcystis aeruginosa* through membrane diffusion has been shown to inhibit the allelopathic activity of the bacteria on the green algae, compared to the direct mixing method (Dunker et al., 2017).

#### 3.1.7 Dialysis tube

Even though the dialysis system operates under a concept similar to the membrane separation system, it is worth including as a separate co-culturing method since it offers a different level of control over the exchanges between the microorganisms. It is a co-culturing method in which molecules and ions produced by one microorganism can selectively move through a dialysis membrane to the culture medium of the other microorganism, and *vice versa*. The properties of the dialysis membrane, such as its semi-permeability and molecular weight cut-offs, determine the size of the molecules that can be transferred or exchanged between the microorganisms. In this method, the guest strain of the microorganism is placed in a dialysis bag and suspended in a large vessel containing the host strain of the microorganism in a free liquid medium. Dialysis-mediated co-culture has been used to explore new interspecies allelopathic interactions with methods such as biochemical analyses, proteomics, and metabolomics. For example, the co-culture of *M. aeruginosa* with *C. vulgaris* through dialysis mediated a negative inhibition of microalgae growth by releasing linoleic acid, while the nitric oxide from *C. vulgaris* stimulated the positive feedback mechanism of linoleic acid production by *M. aeruginosa* (Song et al., 2017).

#### 3.1.8 Agar

Agar systems offer a method of spatially separating and co-culturing microorganisms on a porous solid agar support with different compositions, such as potato dextrose and LB-agar (Barka et al., 2002). While this might resemble the process of flocculation, it is essential to differentiate that the cell aggregation in agar systems happens due to the solid medium’s properties rather than the induced properties of the microorganisms. The concentration of agar used may vary depending on the nature of the microorganisms and the purpose of the study. Co-culturing *Pseudomonas diminuta* and *Pseudomonas vesicularis* on agar plates with *Scenedesmus bicellularis* and *Chlorella* sp. Revealed that the rate of diffusion for info-chemicals depends on the agar’s volume, porosity, and composition (Mouget et al., 1995). The extracellular metabolites in a large pool of culture medium with a very low concentration are difficult to isolate, identify, quantify, and reproduce.

In conclusion, these methods indeed have overlapping features, but it is the nuanced differences and the contexts in which they are used that create their individual identities. From the simplicity and cost-effectiveness of direct mixing to the unique control mechanisms of encapsulation and dialysis tube systems, each method has its own specific applications and benefits.

#### 3.1.9 Applications, advances, challenges, and future prospective in microalgae-bacteria co-cultivation

**FIGURE 4:**
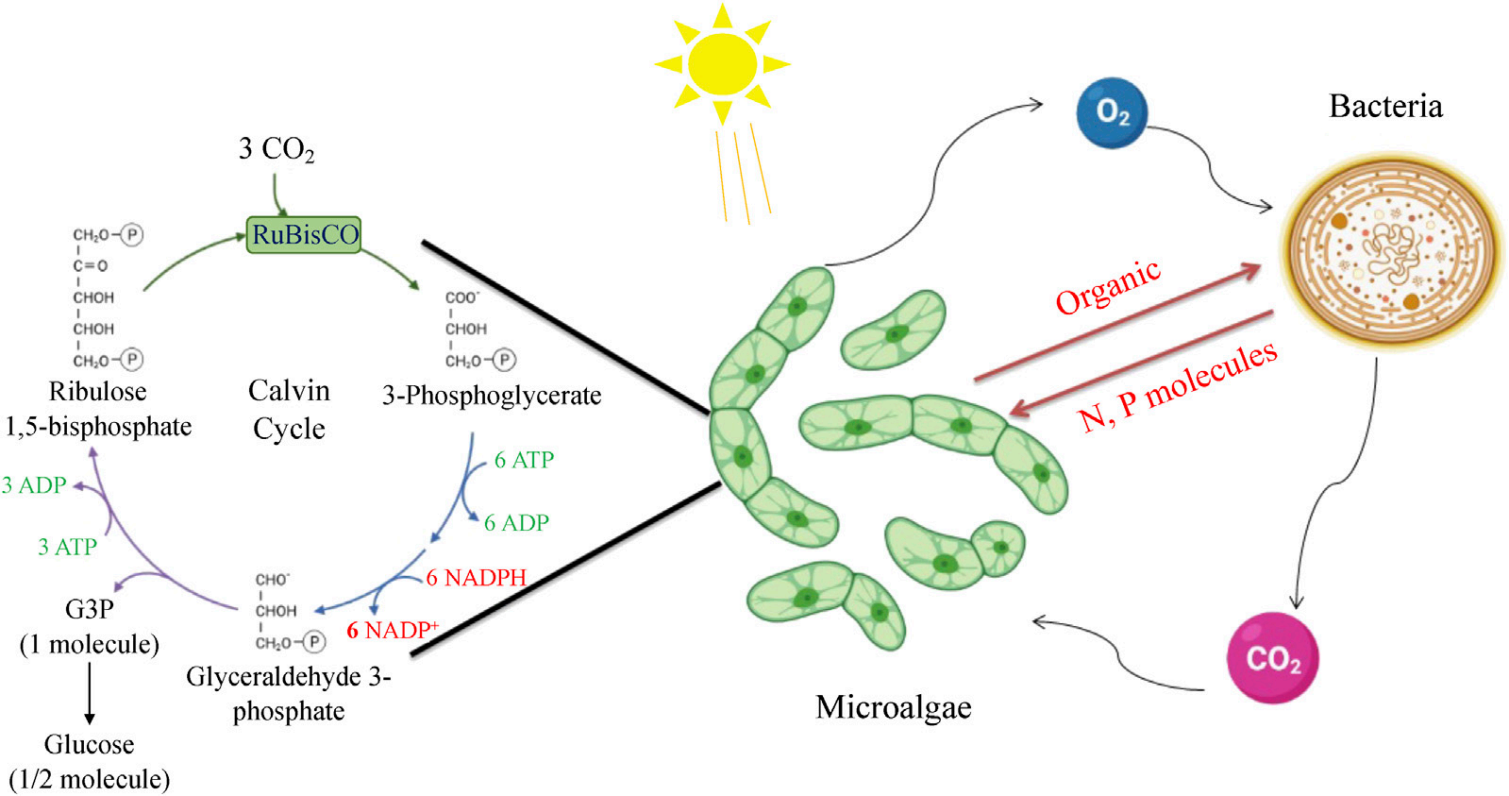
Metabolic interactions of microalgae and bacteria in the co-culture system.

Microalgae have been extensively studied for co-culturing with growth-promoting bacteria. This is due to their ability to produce extracellular chemicals and act as a potential and ecologically sound alternative to current carbon sequestration techniques for CO_2_ mitigation. Although most microalgae lack the mechanism for fixing nitrogen and phosphorus, they compensate by providing fixed organic carbon to the bacteria (Kim et al., 2014) (Figure 4). This cooperative interaction has proved to be an effective co-culturing method for microalgae and bacteria, with increasing densities of bacteria and microalgae expected to strengthen the beneficial relationship. In the context of CO_2_ bio-mitigation applications, microalgae-bacteria consortia can supply O_2_ and organic compounds for bacterial consumption through photosynthesis (C3 Calvin cycle and C4 pathways), while bacteria can produce CO_2_ and inorganic substances to support microalgal growth (Liu et al., 2017). By utilizing this mutually beneficial interaction of CO_2_ and O_2_, the capital expenses for the oxygenation of activated sludge tanks and the risk of thermal decomposition can be significantly decreased (Acién et al., 2016). Furthermore, bacteria can produce micronutrient metabolites such as vitamin B12, phytohormones, thiamine derivatives, and siderophores to speed up microalgal metabolism and biomass growth (Ramanan et al., 2016). The interactions between microalgae and heterotrophic bacteria also occur in oligotrophic conditions, particularly through macronutrient-mediated interactions.

The use of microalgae-bacteria symbiosis for bioremediation offers several advantages over conventional methods, such as its ability to withstand a variety of environmental conditions, the stability of the partnership, metabolite and nutrient exchange, and protection against invading species (Subashchandrabose et al., 2011). For example, the co-cultivation of *C. vulgaris* with activated sludge bacteria improved nutrient and dissolved oxygen performance and facilitated microalgal harvesting (Medina and Neis, 2007). The remediation of synthetic wastewater was also enhanced by the co-cultivation of photosynthetic bacterium *R. sphaeroides* and green algae *C. sorokiniana* (Ogbonna et al., 2000). The interactions between microalgae and bacteria have been found to enhance microalgal biomass production and nutrient removal (Sültemeyer, 1998). Careful selection of co-culture members is important for efficient nutrient removal from wastewater and enhanced algal biomass production. Table 1 provides an overview of microalgae-bacterial co-culture systems used for bioremediation, biomass, and lipid production.

In biofuel production, microalgae-bacteria co-cultures have demonstrated improved efficiency compared to monocultures. Co-cultures have been successfully used to convert microalgae biomass into various types of biofuels, including biodiesel, biohydrogen, bioethanol, biomethanol, biobutanol, and biomethane, using lipids, carbohydrates, and proteins (Owolabi et al., 2012). Biodiesel is a promising renewable energy source that can significantly reduce emissions of unburned hydrocarbons and carbon monoxide, without the production of sulfur and aromatic byproducts (Mondal et al., 2017). Microalgae can produce organic matter, such as triglycerides (TAGs), from CO_2_ and water, which can be used as precursors for biodiesel production (Scott et al., 2010). TAGs are usually over-accumulated and stored in specialized lipid bodies present in the cytosol of the cells (Mata et al., 2010). Some species of *Pseudomonas* in association with *Chlorella* sp., have been efficiently utilized for biodiesel production (Bell et al., 2016).

Moreover, microalgae have the potential to be a renewable and carbon-neutral source of energy through the production of hydrogen fuel, which can be converted into electricity for various applications. Bacterial fermentation produces CO_2_, which promotes microalgal growth; on the other hand, oxygen is synthesized during microalgal photosynthesis, promoting bacterial growth. However, due to the production of oxygen and the presence of hydrogen, which make an explosive gas combination, the removal of oxygen from the media, through physical or chemical methods, is a critical step in achieving efficient H_2_ photoproduction (Fakhimi et al., 2020). Among the various H_2_ photoproduction systems studied for yield and sustainability, *Chlamydomonas* in consortia with different bacterial species such as *Pseudomonas*, *Bacillus*, *Clostridium*, *Bradyrhizobium japonicum*, *Rhizobium etli*, and *Escherichia coli* have been extensively researched (Xu et al., 2016; Ban et al., 2018). Microalgae-bacteria consortia, specifically the *Chlamydomonas*-bacteria system, have shown potential for enhancing algal H_2_ production through starch accumulation and metabolite exchange secretion. This process involves the release of electron donors such as acetate, ethanol, formate, and glycerol into the medium, which can be used by bacteria to synthesize H_2_ (Xu et al., 2017; Carbonell et al., 2018). Additionally, *Lactobacillus amylovorus* can hydrolyze starch to lactic acid from algal biomass, which can be used for photo-H_2_ production in *Rhodobacter capsulate, Rhodobacter sphaeroides, Rhodobium marinum, and Rhodospirillum rubrum* (Kawaguchi et al., 2001). Some bacteria can also synthesize H_2_ through fermentative pathways. Certain bacteria, such as *Clostridium*, have a PFOR-H_2_ production pathway that can synthesize acetic acid as an end-product. Similarly, *E. coli* uses FPL-H_2_ production pathways to obtain acetic acid and ethanol as end products (Oh et al., 2011).

The potential of microalgae-bacteria symbiosis for bioethanol production from polysaccharides such as starch, cellulose, and sugars through fermentation has also gained attention. Microalgae species like *Chlorella, Dunaliella,* and *Scenedesmus* have been identified as feedstock for bioethanol production due to their high starch content (Özçimen et al., 2015). Starch granules make up to 40% of most microalgae species’ dry weight, and bacteria can ferment these to produce ethanol (Ramanan et al., 2016). Marine microalgae’s starch-containing biomass can be saccharified to produce ethanol using amylase from the bacterium *Pseudoalterimonas undina* (Matsumoto et al., 2003). Efficient bioethanol production has been demonstrated by enzymatic hydrolysis of *C. reinhardtii* using amylase from the marine bacterium *Bacillus licheniformis*, followed by fermentation with *Saccharomyces cerevisiae* (de Farias Silva and Bertucco, 2016).

Microalgae provide trace elements like iron, cobalt, and zinc that can fulfill the nutrient requirements for bacteria, whose biomass can be converted into biomethane and biogas by anaerobic digestion (Grobbelaar et al., 2004). The metabolic activities of anaerobic bacteria can impact the proportion of proteins, carbohydrates, and lipids in the biomass (Illman et al., 2000), and the methanogenic potential of microalgae can be influenced by the protease resistance of their cell walls (Angelidaki and Sanders, 2004). The lysis of microalgal cell walls in *Botryococcus braunii* and *Nannochloropsis gaditana* can be achieved by endoglucanase activities of various cellulolytic bacterial species (Muñoz et al., 2014)*.* Bio-augmentation of *C. vulgaris* biomass with a cellulolytic and hydrogenogenic bacterium, *Clostridium thermocellum,* can improve the degradation efficiency, leading to higher levels of methane and hydrogen production and increasing the overall biogas yield (Lü et al., 2013).

Microalgae biomass is composed of carbohydrates, proteins, lipids, and organic and inorganic molecules that can be converted into various products through enzymatic, chemical, or microbial conversions (Mutanda et al., 2020). Microalgae are rich in poly-unsaturated fatty acids (PUFAs), such as arachidonic acid, docosahexaenoic acid, linolenic acid, and eicosapentaenoic acid, which have potential applications as dietary supplements for humans and animals (Benemann, 1990; Radmer, 1996). The β-carotene from the microalgae *Dunaliella salina* is used as an antioxidant supplement in humans and animals (Spolaore et al., 2006), while microalgal biomass from *Chlorella*, *Dunaliella*, *Isochrysis*, *Nannochloropsis*, *Nitzschia*, *Pavlova*, *Phaeodactylum*, *Scenedesmus*, *Skeletonema*, *Spirulina*, *Tetraselmis*, and *Thalassiosira* have been used as feed for mollusks, crustaceans, and fish. Additionally, microalgal biomass can be used to produce fertilizers (Metting and Pyne, 1986), exopolysaccharides for medical and pharmaceutical purposes (Zhang et al., 2019), as well as biodegradable plastics, bioflocculants, bioactive compounds, cosmetics, and polysaccharides (Olaizola, 2003; Pulz and Gross, 2004).

The current drawbacks of microalgae-bacteria co-cultivation include its high cost and low sustainability due to challenges associated with its disposal. However, recent research suggests that the use of biomaterials with advanced harvesting methods can facilitate the repurposing of residual organic matter for the production of biofuels, biomolecules, and animal feed, thus promoting a more sustainable bio-economy (Stiles et al., 2018). Microalgae-bacteria symbiosis has gained attention as a cost-efficient method for wastewater treatment and bioremediation, as it enables successful removal of pollutants, sequestration of greenhouse gases, flocs production, and elimination of pathogens (Barati et al., 2021a; Barati et al., 2021b; Saravanan et al., 2021).

## 4 Microalgae fungi co-cultivation

Microalgae-fungi co-cultivation despite being a more recent method in comparison to microalgae-bacteria co-culture, offers promising outcomes for microalgae biomass separation via co-pelletization into fungal pellets. This process utilizes filamentous fungi-based flocculation, an approach that is cost-effective and environmentally-friendly, given its lack of chemical reliance, ability to yield various floc sizes, and wide applicability in biomass processing (Xie et al., 2013; Rosero-Chasoy et al., 2021). The interplay between fungi and microalgae within a shared ecological niche involves competition for nutrients and space, with the release of extracellular enzymes that hold potential for the production of high-value products (Sandland et al., 2007; Bertrand et al., 2014). Antagonistic (where fungi benefit from the host), mutualistic (where both organisms benefit from one another), and parasitic (where fungi benefits from other microorganisms) relationships often typify the dynamics between fungi and other microorganisms (Yu et al., 2021). The process of fungal pellet formation involves the germination of embryonic mycelium germinates from the fungal spore branch (fungal hyphae) leading to visible pellets. When growth conditions deteriorate, the hyphae begin self-decomposition and can aggregate in submerged culture (Espinosa-Ortiz et al., 2016). Pellet formation is dependent on electrostatic interactions, hydrophobicity, and the specific interactions of spore wall components, influenced by the medium composition and physicochemical properties of fungi (Grimm et al., 2005; Zhang and Zhang, 2016). Microalgae-fungi co-cultivation under optimal conditions can lead to the formation of fungi-microalgae pellets, facilitating microalgae harvesting through simple filtration, a fundamental principle behind this method. The fungal spore-assisted (FSA) or fungal pellet-assisted (FPA) methods are commonly employed for microalgal harvesting from the co-culture of microalgae with fungal spores or pre-cultured fungal pellets (Chen et al., 2018; Yang et al., 2019). Microalgae cells can bind to fungal cells, possibly via a charge-neutralization mechanism (Zhang and Hu, 2012; Wrede et al., 2014). The negative charge on the algae surface at neutral pH due to the presence of proton-active carboxylic, phosphoric, phosphodiester, hydroxyl, and amine functional groups, can be neutralized by the positive charge on fungi, acting as a cationic flocculant towards microalgae (Wrede et al., 2014). The mutual benefits of co-cultivation of microalgae and fungi, particularly yeast, has been evidenced in biodiesel production, wastewater treatment, chemical production, and aquaculture feed applications. For instance, yeast in a co-culture system can generate carbon dioxide for microalgae biosynthesis, while microalgae provide oxygen for yeast respiration. The efficiency of resource utilization and reduction of carbon emissions into the atmosphere, however, are offset by challenges in biomass production cost and energy-efficiency (Rakesh and karthikeyan, 2019).

### 4.1 Microalgae-fungi co-cultivation methods

Co-cultivation techniques can vary, each with distinct benefits and limitations. These methods include direct mixing, encapsulation, pelletization and flocculation, biofilms, and solid-liquid interfaces. Notably, it is important to understand the nuances between seemingly similar techniques, such as membranes and dialysis, or agar and flocculation, each having specific applications and effects on co-cultivation. While these methods can generally improve efficiency compared to monocultures, it is critical to validate this claim with empirical evidence from specific applications.

#### 4.1.1 Direct mixing

This method, whereby microalgae and fungi are co-cultivated resulting in direct interaction and exchange of signaling molecules within the same environment (Brenner et al., 2008), is common to many co-cultures. It has been utilized to explore the physical and biochemical interactions, yield parameters, and metabolite production between fungi and algae (Oh et al., 2007). For example, in the co-culture of *Chlorella* and *Aspergillus,* microalgae biomass yield, lipid content, and cellular oil exhibited improvements (Yang et al., 2019). Similarly, the consortium of *Mucor circinelloides* and *C. vulgaris* showed improved biomass yield, lipids, and saturated and unsaturated fatty acids that can be utilized for biodiesel production (Zorn et al., 2020). However, the effectiveness of this method significantly depends on the selection of microorganisms and their interactions. For example, establishing a balanced co-culture of *Scenedesmus obliquus* and *Candida tropicalis,* by altering the population density to algae: fungi (2:1), improved algal biomass production (Wang et al., 2015). The significance of inoculation ratios/population density was later confirmed by a study using a consortium of *Chlorella pyrenoidosa* and *Rhodotorula glutinis*, where a higher ratio of algae: fungi (3:1) was found to be ideal for achieving the highest biomass concentration and lipid productivity as well as enhancing nutrient removal from wastewater and protein productivity (Li et al., 2019). Direct mixing of the microalgae *Chlorella* sp. and *S. cerevisiae,* showed an increase in biomass and lipid productivity of *Chlorella* sp. and enhanced the carbon bio-fixation compared to their mono-culture (Shu et al., 2013). Furthermore, co-cultivation of *S*. *obliquus* and *R*. *glutinis* showed a significant increase in the biomass and lipid productivity of *S*. *obliquus* (Yen et al., 2015). This method, though commonly used, does not apply to all co-cultures and variations exist based on the organisms and environment used.

#### 4.1.2 Encapsulation

This method, where microorganisms are immobilized through gel entrapment, usually involves natural polysaccharides such as alginates and agar (Kitcha and Cheirsilp, 2014). This is distinct from pelletization and flocculation, as encapsulation focuses on entrapment within a gel matrix. For instance, co-capsulation of the yeast *Trichosporonoides spathulata* and the microalgae *C. vulgaris* in alginate gel beads not only simplifies the harvesting process but also maintained growth and lipid production of *C. vulgaris* at levels comparable to those of free cells (Kitcha and Cheirsilp, 2014).

#### 4.1.3 Pelletization and flocculation

Co-cultivation of microalgae with fungi can lead to natural pelletization and flocculation. For example, co-cultivation of *Chlorella protothecoides* and *Tetraselmissuecica* with fungal strains isolated from compost, straws, and soil resulted in higher biomass, lipid productivity, and bioremediation efficacy compared to monocultures (Muradov et al., 2015). Similar co-cultures of *C. vulgaris* and two species of *Aspergillus* sp. Xhibited similar results (Zhou et al., 2012). *Pleurotus ostreatus,* an edible fungus strain, has been developed to increase the efficiency of microalgae harvesting for feed or food production in a low-cost manner (Luo et al., 2019). They found that pellets of *Pleurotusostreatus* co-cultured with *Chlorella* sp. at 100 rpm agitation and low pH showed better harvesting efficiency than pellets cultured under 0 rpm and 150 rpm agitation. In heterotrophic co-culture conditions, the co-culture of *Aspergillus niger* and *C. vulgaris* presented lower flocculation efficiency compared to autotrophic conditions (Zhang and Hu, 2012). The pelletization and flocculation efficiency of the consortium can be influenced by the co-cultured microorganisms and the carbon source used in the system (Gultom et al., 2014; Luo et al., 2019). The optimal culture conditions for pelletization and flocculation can vary depending on the system. For instance, co-cultivation of filamentous fungus (*A. niger*) and microalgae (*C. vulgaris*) to produce cell pellets was evaluated under various concentrations of organic carbon sources (glucose, glycerol, and sodium acetate), and the optimal culture conditions for reaching >90% cell harvest efficiency were found to be 2 g L^-1^ glucose as an organic carbon supply for fungal growth and the formation of cell pellets (Gultom et al., 2014). However, the concentration of the flocculant and its binding strength were proven ineffective at higher microalgae biomass concentrations, resulting in variations in pellet morphology. A co-culturing ratio of 1:300 (fungi: microalgae) improved the harvested efficiency by over 90% (Gultom et al., 2014). The co-pelletization of autotrophic microalgae *C. vulgaris* by precultured *Aspergillus oryzae* pellets promoted biomass production (99.23%), lipid (33.97%), and biofuel production (Chu et al., 2021). Although charge neutralization was not the main mechanism involved in fungi-algae aggregations, changes in functional groups on cell surfaces and secreted metabolites in the medium could be mainly responsible for inducing the bioflocculation process.

#### 4.1.4 Biofilms

In the field of bioremediation and bioprocessing applications such as biomass harvesting, mycoalgae biofilms, resembling lichen structures on a supporting polymer matrix, have been gaining interest. The concept of mycoalgae biofilms has stemmed from previous knowledge of fungi and algae interactions (Rajendran and Hu, 2016; Rajendran et al., 2017). Biofilms formed by microalgae in the presence of non-photosynthetic cohabitants, such as *Acremonium* sp. and *Aspergillus* sp., have shown enhanced biomass growth and photosynthetic efficiency. These improvements have been evidenced by fingerprint profiles of the isolated photosynthetic components (Miranda et al., 2017).

#### 4.1.5 Solid-liquid interface

In a novel approach, a photobioreactor system was used to enhance biomass and lipid productivity of microalgae and yeast in a co-culture (Santos et al., 2013). This system is unique to the co-culture of *Rhodosporidium toruloides* and *C*. *protothecoides* and involves the heterotrophic growth of the former and autotrophic of the latter in separate vertical-alveolar-panel (VAP) photobioreactors. These photobioreactors are connected via the gas phase to enable the exchange of O_2_ produced by microalgae and CO_2_ produced by yeast. Unlike industrial flue gas, which is often associated with toxicity, the CO_2_ produced by yeast was not toxic to the microalgae. The system resulted in a 94% increase in biomass productivity and an 87% increase in lipid productivity of *C. protothecoides* compared to normal cultivation conditions. The uniqueness of this method underlies its distinction from other methods like encapsulation and biofilms, hence it is not universally applicable.

While the aforementioned co-culture methods share similarities, such as the co-cultivation of microorganisms, they each have unique characteristics and applications that differentiate them from each other. Also, while microalgae-bacteria co-cultures have demonstrated improved efficiency in biofuel production compared to monocultures in certain cases, it is important to note that these results can vary significantly based on the organisms used, the environmental conditions, and the specific method employed.

#### 4.1.6 Applications, advances, challenges, and future prospectives of microalgae-fungi co-cultivation

The use of fungi in facilitating microalgae harvesting and wastewater treatment has attracted considerable recognition recently, primarily because of its cost-effectiveness and high efficacy (Leng et al., 2021). Microalgae and fungi can form co-pellets, aiding microalgae harvesting through electrostatic neutralization, protein surface interaction, and exopolysaccharide adhesion, as a result of the co-culture process (Serra et al., 2008; Leng et al., 2021). The interplay of heterotrophic or mixotrophic relationships within this system culminates in elevated Chemical Oxygen Demand (COD) elimination. Through RuBiSCO or similar enzymes, CO_2_ molecules diffuse into microalgae cells and undergo the Calvin-Benson-Bassham cycle (CBB), synthesizing oxygen and other organic matter for metabolic purposes (Gonçalves et al., 2017). The process results in enhanced growth and development of both partners due to the gas exchange, which subsequently reduces the carbon content in the wastewater (Wang Y. et al., 2016). Fungal secretions of extracellular enzymes and the pellet structures they form with microalgae aid in capturing suspended solids (Wang Y. et al., 2016). The microalgae-fungi consortium effectively rids treated wastewater, activated sludge, and biogas slurry of COD and nutrients, outperforming monoculture (Wang et al., 2017; Yang et al., 2019). The co-culture of microalgae *Chlorella* sp. and yeast *S. cerevisiae* aerated by 1% CO_2_ demonstrated an increased CO_2_ bio-fixation rate of 64.75 mg.L^-1^. h^-1^, leading to a significant boost in cell density (X_max_ = 73.7%), and maximum oil production (P_max_ = 93.3%) compared to *Chlorella* sp. Monoculture (Shu et al., 2013). Similarly, the co-cultivation of *C*. *protothecoides* and yeast *Rhodosporidium toruloides* in a VAP photobioreactor had a CO_2_ bio-fixation rate of 29 mg.L^-1^. h^-1^, nearly twice that of the control cultivation in a VAP photobioreactor (Santos et al., 2013).

The joint cultivation of microalgae and fungi outperforms mono-microalgae and mono-fungi systems in phosphorus removal (Yang et al., 2019). For instance, the co-culture of *C. vulgaris* and *Ganoderma lucidum* demonstrated a higher efficiency in phosphorus removal from wastewater (Zhou et al., 2012). The pH reduction from the microalgae/fungi co-culture and fungi´s enzyme secretions facilitate the degradation of precipitated PO_4_-P and promote phosphorus assimilation (Zhang et al., 2020). Nitrogen removal is also considerably enhanced when filamentous fungi are co-cultured with microalgae (Zhou et al., 2012). For example, the combination of fungi and microalgae in municipal water resulted in a 100% removal efficiency of NH_4_-N within a day (Salih, 2011). The nitrogen exchange in co-culture systems between fungi and microalgae was established with isotopic labeling experiments (Du et al., 2019). Co-cultivating microalgae *Scenedesmus* sp. and wild yeast (1:1 v/v) achieved high nutrient removal rates (96% nitrate, 100% total ammonia nitrogen, and 93% orthophosphate) (Walls et al., 2019). The microalgae/fungi cell wall comprises functional groups such as cellulose, hemicellulose, protein, and other polymers with excellent adsorption properties, electrostatic interactions, ion exchange, and chelation/complexation, making them useful for eutrophication of heavy metals, drug, and antibiotic removal (Leng et al., 2021). The co-culture pellet of microalgae/fungi proved more efficient in absorbing arsenic and gold than monocultures (Bodin et al., 2016; Shen and Chirwa, 2020). Additionally, the fungi-assisted microalgae harvesting process shows promise in the removal of a wide range of pesticides in wastewater (Hultberg and Bodin, 2018).

The combined use of microalgae and fungi has considerable potential for the industrial production of biofuels, including biodiesel, bioethanol, biomethane, and biohydrogen (Prajapati et al., 2016). Lipids extracted from *S*. *obliquus* and *Cunninghamella chinulata* pellets have been shown to improve the fuel properties to meet international standards (Srinuanpan et al., 2018). Also, flocculation *of N. oceanic* with oleaginous fungi *Mortierella elongata* enhances the yield of poly-unsaturated fatty acids (PUFAs) (Du et al., 2018). However, the composition and yield production of lipids and fatty acids can vary significantly depending on the fungi and microalgae strains combined in the consortium. The classes of lipids generated through these consortia could be modulated, and fatty acid composition could be tailored and optimized by co-culture parameters, which is beneficial for biodiesel production (Leng et al., 2021) (Table 2).

**TABLE 2:** Microalgae-fungal co-culture systems used for bioremediation, biomass, and lipid productions (T-temperature).

Microalgae/fungus	Product	Culture method	Yield	Reference
*C. protothecoides/A. fumigatus*	Biodiesel/fuel, biomass, lipid production, bioremediation	Microtitre plates, T- 25°C, Time- 48 h, light 200 μmol m^−2^ s^−1^	Increased biomass production, lipid yield, wastewater bioremediation efficiency	Muradov et al. (2015)
*C*. *vulgaris/A*. *niger*	Enhance microalgae harvest	Potato dextrose broth (PDB) medium, T-28°C, pH 7.5,Time- 6–7 days	Improve flocculation activity and biomass production	Carruthers et al. (2017)
*C. vulgaris/G. lucidum*	Bioremediation, biogas purification	Erlenmeyer flasks, BG-11 medium, T- 25°C ± 0.5°C,Time- 7 days, light:dark cycle- (12 h:12 h),light- 200 μmol m^−2^ s^−1^	COD, N, P and CO_2_ removal efficiency 68.29%, 61.75%, 64.21%, 64.68%, respectively	Zhou et al. (2018)
*Microalgae/A. fumigatus*	Biomass production, bioremediation	Erlenmeyer flasks, liquid fungal growth broth (FGB) medium, T- 28°C, Time- 48 h, light- 200 μmol m^-2^s^-1^	Increased biomass production, lipid yield, and wastewater bioremediation efficiency	Wrede et al. (2014)
*Chlorella* sp.*/Aspergillus* sp	Biomass, lipid, biodiesel production, bioremediation	Erlenmeyer flasks with 100 mL actual wastewater	Biomass yield (4.215 g/L), lipid content (35.2%), microbial cell oil with a lower unsaturation degree	Yang et al. (2019)
*Scenedesmus* sp.*/T*. *reesei*	Bioremediation	Conical flask, light intensity- 3,500 lux, T- 30°C ± 2°C, Time- 7 days	COD, N,P removal were >74%, >44%, and >93%, respectively	Srinuanpan et al. (2018)
*N*. *oceanica/M. elongata*	Biofuel productivity engineering of poly-unsaturated fatty acids (PUFAs)	light:dark cycle- 14 h:10 h, T- 23°C	High levels of TAG and total fatty acids, PUFAs	Carbonell et al. (2018)
		light- 0–2000 μmol m^−2^ s^−1^		

Biomass production and lipid yield from microalgae/fungi co-culture are higher than the mono-culture, and the exchange of gases and nutrients, enhances individual metabolic activity, enabling the consortium to accumulate nutrients from the surroundings more effectively (Piercey-Normore and Athukorala, 2017). Fungi can use carbon resources stored in microalgal cell walls by using various extracellular enzymes such as fat hydrolase and cellulolytic enzymes to directly synthesize free fatty acids for cell growth and proliferation (Lennen and Pfleger, 2013). Additionally, fungi can convert the adsorbed fatty acids into Triacylglycerides and accumulate them into lipid bodies (Ji and Ledesma-Amaro, 2020). As the microalgae biomass reaches a specific concentration, the shading effect usually restricts autotrophic microalgae from getting sunlight. The microalgae cells, fixed in pellets in the microalgae/fungi consortium, can facilitate light transmission, promote the overall growth of microalgae, and significantly increase the algal biomass yield (Prajapati et al., 2016). Although microalgae/fungi co-culture biomass shows considerable potential for producing value-added products, biomass harvesting, and separation can be challenging due to the hydrolyzation of microalgae cell walls through the interaction between microalgae and fungi. This situation is not ideal for separately recovering microalgae or fungi. Moreover, biomass obtained from wastewater, whether fungal or bacteria-assisted harvesting, should be carefully implemented in applications such as food and feed supplements and cosmetics production.

Co-culture of microalgae and yeast is a promising technology for lipid production due to the mutual benefits conferred by the co-culture of the two microorganisms (Magdouli et al., 2016). Cheirslip et al. demonstrated that the growth and lipid productivity of *R. glutinis* increased when co-cultivated with microalgae *C. vulgaris* in industrial waste (Cheirsilp et al., 2011). In another study, the growth of *S. obliquus* was increased by 30.3% in a co-culture with *C*. *tropicalis*, and its lipid content and productivity were enhanced compared to the microalgae monoculture system (Wang R. et al., 2016). A study on a co-cultivation system of *Chlorella* sp. *KKY-S2* and yeast *Torulaspora maleeae Y30* reached a maximum lipid yield of 1.339 g.L^-1^ after 5 days in the co-culture system, which was higher than the lipid yield (0.969 g.L^-1^) achieved by using atmospheric CO_2_ after 6 days of cultivation (Puangbut and Leesing, 2012). The highest biomass and lipid content of a co-culture system of yeast *Trichosporonoides spathulate* and microalgae *C. vulgaris* in a photobioreactor under optimum conditions reached 12.2 g.L^-1^ and 47%, respectively (Kitcha and Cheirsilp, 2014). Co-culture of oleaginous yeast *T. maleeae Y30* and *T. globose YU5/2* with microalgae *Chlorella* sp. Resulted in higher growth and biomass concentration than their monocultures (Puangbut and Leesing, 2012). The monoculture of *T. maleeae*, *T. globose*, and *Chlorella* sp. Showed a biomass concentration and lipid yield of 8.267 g.L^-1^ and 0.920 g.L^-1^, 8.333 g.L^-1^ and 1.141 g.L^-1^, and 1.933 g.L^-1^ and 0.052 g.L^-1^, respectively. The biomass concentration and lipid yield of co-cultivation of *T. maleeae* and *T. globose* with *Chlorella* sp. were 8.733 g.L^-1^, 1.564 g.L^-1^, 8.010 g.L^-1^, and 2.424 g.L^-1^, respectively (Papone et al., 2012). Various methods used for biomass, lipid, and protein production and wastewater treatment by microalgae-yeast co-cultivation are mentioned in Table 3.

**TABLE 3:** Microalgae-yeast co-culture systems used for different applications.

Microalgae/yeast	Product/Application	Culture method	Yield	Reference
Desmodesmus sp./*R. kratochvilovae*	Lipid, carotenoid, ergosterol	Bioreactor 3L, T:22°C, Time:144 h	Lipids increase to the final value of 29.62%–31.61%, 5.41–6.09 mg.g^-1^ carotenoid production, 6.69 mg.g^-1^ ergosterol yield	Szotkowski et al. (2021)
*C*.*vulgaris*/*R. glutinis*	Biomass and lipid production	Bubble column photobioreactor 260 mL, 100 mol.m^-2^.s^-1^ light intensity	Biomass yield 17.3% and lipid yield 70.9%	Zhang et al. (2014)
*C. pyrenoidosa*/*R. glutinis*	Biomass and lipid production	250 mL Erlenmeyer flask	Biomass concentration 6.12, lipid yield 2.48, total fatty acid productivity 2 fold than in the monoculture	Liu et al. (2018)
*S*. *obliquus*/*R. glutinis*	Biomass and total lipid	5 L photobioreactor	40%–50% biomass increased and 60%–70% total lipid increased	Yen et al. (2015)
*C. zofingiensis*/*X. dendrorhous*	accumulate high-value astaxanthin and lipid	100 mL Flask	Maximum astaxanthin and lipid yield achieved 5.50 mg.L^-1^ and 2.37 g.L^-1^, respectively. Which were 1.10- and 2.72-fold compared to*C. zofingiensis* monoculture	Jiang et al. (2018)
*C*. *pyrenoidosa*/*R*. *toruloides*	Lipid production and TN and total phosphorous removal	150 mL flask, 2000 lux, 12:12 h light and dark cycles	Lipid content and lipid yield achieved were 63.45% ± 2.58% and 4.60 ± 0.36 g.L^-1^. Total nitrogen (TN), and total phosphorous (TP) at 95.34% ± 0.07%, 51.18% ± 2.17%, and 89.29% ± 4.91%, respectively	Ling et al. (2014)
*C*. *pyrenoidosa*/*R. glutinis*	Protein production and wastewater treatment	1000 L bioreactor	Achieved the removal efficiencies of 58.53%, 36.07%, 33.20%, and 56.25% for ammoniacal nitrogen (NH3-N), total nitrogen (TN), total protein (TP), and chemical oxygen demand (COD), respectivel, and gained 59.8% (w/w) protein	Li et al. (2019)
*Desmodesmus* sp./*R. kratochvilovae*	Biomass, and lipid production	3L bioreactor	8.78–11.12 g.L^-1^ of dry biomass, 29.62%–31.61% lipid content	Szotkowski et al. (2021)
*C. vulgaris/R. glutinis*	Biomass and lipid production	250 mL flask, 6.0 Klux light intensity, T: 26 °C	Biomass production was increased compared to mono-culture, lipid content was 5 times greater than mono-cultures of microalgae and yeast	Ashtiani et al. (2021)

Yeast and microalgae can mutually benefit each other in a co-culture system by using the O_2_ generated by microalgae and the CO_2_ and organic acids produced by yeast. However, some of these organic acids can inhibit yeast growth. Yeast can also provide microalgae with simple sugars obtained by breaking down complex sugars like low-cost agricultural waste, reducing the total cost of the final product (Rakesh and karthikeyan, 2019).

Both yeast and microalgae are promising feedstocks for biodiesel production due to their high lipid content, but the high operational cost and low lipid productivity make current industrial biodiesel production using microalgae or yeast impossible (Meng et al., 2009). Various strategies, including nutrient starvation, multi-stage cultivation, genetic engineering, and co-cultivation, have been implemented to address these issues (Arora et al., 2019; Esakkimuthu et al., 2020). However, nutrient starvation leads to a significant reduction in biomass production, markedly reducing microalgae’s lipid productivity. Additionally, genetic engineering requires extensive knowledge about the compartmentalization of photosynthesis, TAG synthesis and regulation, and the connection between TAG synthesis (Esakkimuthu et al., 2019).

A mixed-culture system of *Chlorella* sp. and *S. cerevisiae* exhibited significant improvements in growth and lipid accumulation with a 128.1% and 165.2% increase, respectively, compared to their respective monocultures. Moreover, the CO_2_ removal rate was enhanced by 195% compared to the *Chlorella* sp. Monoculture (Shu et al., 2013). Similarly, a mixed-culture of *Isochrysis galbana* and *Ambrosiozyma cicatricosa* showed higher growth rates and biomass concentration than their monocultures (Cai et al., 2007). The co-cultivation of yeast *R. glutinis* and microalga *Spirulina platensis* demonstrated a lipid yield of 467 mg.L-1, which was 3.18 times higher than yeast monoculture and 3.92 times higher than microalgae monoculture (Xue et al., 2010). These findings demonstrate that the co-culture of microalgae and yeast can lead to improved growth and lipid accumulation, highlighting its potential for sustainable biofuel production.

In wastewater treatment, the presence of high levels of organic suspended solids (COD>5 g.L^-1^) and turbidity can impede microalgae growth and limit its performance. However, this issue can be addressed by co-cultivating microalgae with heterotrophic microorganisms like yeast. For instance, co-cultivating *R. glutinis* and *S. obliquus* led to a 40%–50% increase in suspended organic solids removal from domestic wastewater (Li et al., 2019). Yeast can thrive in a medium with high COD concentrations (ranging from 15 to 50 g.^L-1^) but may not efficiently remove nitrogen and phosphorous. Therefore, co-cultivating microalgae and yeast can lead to the efficient removal of COD, total organic carbon (TOC), nitrogen, and phosphorous from wastewater (Ling et al., 2014). Additionally, the co-cultivation of *C. vulgaris* and *Yarrowia lipolytica* in a liquid digestate from the yeast industry demonstrated better growth and nutrient removal compared to monoculture systems of each microorganism (Yang et al., 2019).

The emergence of the microalgae-fungi consortium is a promising strategy for bioremediation, microalgae biomass, lipids, and biofuel production. However, this technology is still in its infancy, and upscaling it for industrial applications poses significant challenges. The choice of microalgae and fungi species for the co-culture, and the conditions of co-culture, such as light intensity, carbon source, and agitation, can greatly influence the entire process, thereby impacting its scalability. To optimize the large-scale cultivation of the microalgae/fungi consortium, additional research using advanced metabolomics or proteomics techniques is required. These techniques could aid in the selection of suitable strains and identification of the underlying mechanisms of interactions between microalgae, fungi, and other microbes in the consortium. The three-way interaction among these microorganisms remains unclear and should be taken into account to achieve the success of the overall process.

It is crucial to continue exploring the synergistic interactions between microalgae and fungi, and to further investigate the potential of these co-cultures for large-scale applications. Despite the present hurdles, the potential benefits of such systems for environmental remediation, renewable energy production, and the sustainable management of resources are substantial. With continued research and development, the prospects for fully realizing the potential of microalgae-fungi consortium are promising.

## 5 Microalgae-microalgae co-cultivation

The majority of existing commercial microalgae cultivations prioritize large-scale growth of single species over co-cultivation of microalgae. However, microalgae monocultures, particularly those in open pond systems which offer greater economic feasibility, are at risk of contamination from other algae, pathogens, and grazers. Contamination control methods, such as pesticide application require a comprehensive understanding of the pests and pose both environmental risks and substantial costs. Co-cultivation of microalgae mitigates these challenges, offering advantages for large-scale microalgae cultivation, including increased stability, resource utilization efficiency, and enhanced biomass, and lipid productivity (Novoveská et al., 2016).

### 5.1 Microalgae-microalgae co-cultivation methods

#### 5.1.1 Direct mixing

As a fundamental method in mixed-culture systems, direct mixing demonstrates considerable potential. The mixed cultivation of *C. sorokiniana* and *Euglena gracilis*, for instance, under photoautotrophic, mixotrophic, and heterotrophic conditions, resulted in notable enhancements to the growth rate compared to monoculture. A total yield of 51.73×10^5^ cells/mg glucose and 67.64 ×10^5^ cells/mg glucose was achieved in the co-culture of the 2 cell strains (Friday et al., 2010).

The co-culture of two selected algae strains, *C. vulgaris* and *Pseudokirchneriella subcapitata*, resulted in increased production of chlorellin, mainly composed of a C18 fatty acid mixture (DellaGreca et al., 2010). Recycling a portion of the harvested biomass to a co-culture system of microalgae promoted the rapid settling of dominant microalgae species, thus increasing biomass productivity and harvesting of the culture by 35% and 25%, respectively (Park et al., 2011). Employing gravity recycling using *Pediastrum boryanum* (a rapidly settleable alga) in the pilot-scale HRAPc system (high-rate algal ponds) for treating domestic wastewater dominated with *Dictyosphaerium* sp. (a poorly settleable alga), improved harvesting and biomass yield (Park and Craggs, 2014). Recycling of either solid or liquid portions had a similar effect on harvest, which may be attributed to the presence of EPSs in the liquid portion of the culture, enhanced cell concentration and efficiency in solar consumption by increasing algal residence time, and/or increased overall growth rate of microalgae due to shifts in the relative proportions of algal growth stage (Park and Craggs, 2014). In another experiment, Park and Craggs studied the effect of different recycling rates (1%, 2.5%, 5%, 10%, 25%, and 50%) on the growth and settling ability of co-cultivating of microalgae in a high-rate algal pond (HRAPs) for treating domestic wastewater. Their results showed that recycling 10% of daily harvested biomass increased biomass productivity and settling ability by 10% and 40%, respectively, contributing to enhancing the harvesting of the culture *Pediastrum boryanum* because of its morphology, improving its concentration by 30% in the medium (Park and Craggs, 2014).

Injection of CO_2_ into microalgae co-culture systems can provide various benefits, such as preserving the microalgae culture from high pH inhibitory effects, enhancing the availability of orthophosphate and ammonia for microalgae utilization, increasing the carbon availability and C:N ratio in wastewater, enhancing the microalgae cells concentration, and increasing the lipid content while improving the lipid profile of the microalgae (Mehrabadi et al., 2017). Optimization of CO_2_ injection and medium pH can also increase the dominance of the suitable microalgae strain for harvesting (Park et al., 2013). For instance, a study performed on co-culturing *S*. *obliquus* and *C. vulgaris* in a flat plate photobioreactor for urban wastewater treatment reported that CO_2_ addition and biomass recycling increased biomass productivity, the dominance of larger microalgae (*S. obliquus*), and gravity sedimentation by 314%, 38%, and 85%, respectively (Fallahi et al., 2021). Furthermore, Mehrabadi et al. reported that the optimum CO_2_ concentration of 10% maximizes biomass productivity of high-rate algal mesocosms (HRAM) leading to a 50% increase compared to the control without CO_2_ injection. CO_2_ injection also enhanced culture harvesting, with 0.5% CO_2_ HRAM biomass increasing mean 1-h harvest efficiency about five times compared to 2% CO_2_ HRAM biomass, and nearly twice that of 5% CO_2_ HRAM. *Micractinium* sp. was the dominant species in both 5, and 10% CO_2_ HRAM biomasses (Mehrabadi et al., 2017). Sun et al. reported that recycling microalgae biomass (2% and 10%) also increased biomass recovery in an HRAP system from 75% to 89% without recycling to 92%–94% with recycling (Gutiérrez et al., 2016).

#### 5.1.2 Encapsulation

Encapsulation offers an innovative approach for achieving high biomass concentrations and immobilizing microalgal cells. The encapsulation of microalgal cells within hollow polymer shells of rhombohedral shape offers a promising strategy for microbial-cell immobilization and high-biomass-concentration applications. The encapsulation is made possible by embedding microalgae in CaCO_3_ crystals, layer-by-layer (LbL) coating of polyelectrolytes, and removal of sacrificial crystals. Embedding microalgae in CaCO_3_ crystals involves a two-step process consisting of heterogeneous crystal nucleation on the cell surface and subsequent cell embedment by crystal growth. This approach enables micrometer-sized microalgae to be perfectly coated in calcite crystals without altering their rhombohedral shape, owing to the favorable surface properties of the microalgal cells for calcite crystal growth (Kim et al., 2018). The surfaces of these microcapsules can be further coated with gold nanoparticles, Fe_3_O_4_ magnetic nanoparticles, and carbon nanotubes (CNTs), which provide additional functionalities such as light-triggered discharge, magnetic separation, and enhanced mechanical and electrical strength, respectively. This technology represents an innovative and versatile platform for a wide range of bioapplications requiring the immobilization of microbial cells (Kim et al., 2018). A high-throughput screening study of algal community combinations was conducted using microfluidic technology to generate millions of parallel, nanoliter-scale mixed cultures for biomass accumulation estimation trials. The study revealed that combining different algal species could result in either positive or negative interactions leading to increased or reduced biomass production, respectively. For instance, *Ankistrodesmus falcatus* and *Chlorella sorokiniana*, and *C*. *sorokiniana* and *Selenastrum minutum* had improved performance, while *Selenastrum capricornutum* and *Scenedesmus ecornis* showed reduced productivity when co-cultured (Carruthers et al., 2017). The interaction between microbial populations can result in enhanced productivity and decreased community invasibility, which are favorable traits for scalable bioproduction systems. Microfluidic devices may be essential for the efficient and cost-effective discovery of such synergistic communities through rapid, high-throughput screening of microbial combinations (Carruthers et al., 2017).

#### 5.1.3 Biofilms

Microalgae biofilms offer a promising alternative for large-scale microalgae cultivation. For instance, a consortium of *Chlorella*, *Nitzschia*, and *Scenedesmus* species grown on mesh-type materials in an open pond showed improved biomass production and system efficiency. The mesh-type substrates linked to microalgae can also remove residual treated wastewater directly, further enhancing system efficiency. In addition, a simple and cost-effective dewatering method using natural sunlight was successfully implemented for algal biomass, instead of using a freeze-drying method, making it a feasible technique for bulk biodiesel synthesis (Lee et al., 2014). Biochar, a carbonaceous solid support, was investigated as a growth substrate for *Klebsormidium flaccidum* and *Anabaena cylindrica* biofilms cultured on BG11 media. After 20 days of incubation under a 16:8 (light/dark) photoperiod, the dry biomass, total carbon, and nitrogen contents of the cultures with and without biochar were compared, revealing an 80% increase in *A. cylindrica* growth with the inclusion of biochar (Kholssi et al., 2018).

The productivity and cost-efficiency of algal biofuel production can be enhanced by a mixotrophic microalgae biofilm composed of *C. vulgaris* and *Scenedesmus dimorphus*. The mixotrophic microalgae biofilm outperforms autotrophic microalgae biofilms in terms of biomass yield, feedstock quality, lipid accumulation, and ash content; producing 2–3 times higher biomass yield, 2–10 times higher lipid accumulation, and 40%–60% lower ash content (Roostaei et al., 2018). Moreover, the growth activities of microalgae biofilms and productivity of mixotrophic biofilms are significantly influenced by cell-surface properties such as hydrophobicity and roughness and they are substantially correlated in particular with surface hydrophobicity (Roostaei et al., 2018).

#### 5.1.4 Advances, challenges, and future prospective

Co-culture systems of diverse microalgae species have exhibited superior stability, a higher rate of biomass and lipid production, and improved biofuel properties compared to monocultures. Microalgal lipids extracted from these co-cultures present a larger quantity of short-chain unsaturated fatty acids, leading to enhanced biofuel characteristics including iodine number, cetane number, octane number, heating value, and kinematic viscosity (Ishika et al., 2017; Das et al., 2021).

Mixed microalgae cultures also show promise in various wastewater treatment systems due to their capacity for bio-flocculation and efficient biomass harvesting. Qin et al. reported chemical oxygen demand (COD) removal rates of 57%–63% and total phosphorous removal rates of 91%–96% in a microalgae co-culture system, which were higher than those of a monoculture system of *Chlorella* sp. (45% COD, and 87% total phosphorous) (Qin et al., 2016). These cultures are also cost-effective, less labor-intensive, and resistant to contamination, making them an attractive choice for wastewater treatment. In binary microalgae cultures, enhanced cell-cell interactions result in the production of more extracellular polymeric substances (EPS) as a metabolic strategy to adapt to unfavorable conditions, such as nutrient deprivation. However, excessive EPS accumulation can inhibit mass transfer and nutrient fixation, hindering microorganisms from utilizing dissolved CO_2_. Thus, a thoughtful selection of microalgae species for co-cultivation is critical for the success of large-scale applications, ensuring productive wastewater treatment and bioenergy production (Ray et al., 2021). Despite improved biomass and lipid yields in microalgae consortia, further exploration is required to fully understand the symbiotic mechanisms at play and optimize productivity. The incorporation of omics resources and genetic engineering techniques, including gene transformation procedures, mutagenesis, and genome-editing tools in co-cultivation studies, promises to unravel the intricate metabolic pathways that microalgal cells undergo (Kuo et al., 2022).

#### 5.1.5 Application of microalgae-microalgae co-cultivation

Co-cultivation of microalgae species offers exciting prospects for environmental pollutant removal and renewable energy production. The mutualistic interactions among microalgae enhance nutrient removal capacity and facilitate adaptation to varying environmental conditions in the wastewater treatment system (Han et al., 2021). For instance, Prathima Devi et al. achieved a biomass concentration of 0.98 mg.L^-1^. d^-1^ by heterotrophic co-cultivation of microalgae collected from an Indian lake in domestic wastewater (Devi et al., 2012). Similarly, in a study by Taskan et al. the organic matter (OM) and nutrient removal efficiencies were investigated by co-cultivating microalgae in a slaughterhouse wastewater treatment photobioreactor. The study reported 70.2%, 96.2%, and 89.6% removal efficiencies for total nitrogen (TN), total phosphorous (TP), and total organic carbon (TOC), respectively (Taşkan, 2016).

Microalgae co-cultivation also provides a potential solution to the high operational costs associated with biodiesel production (Zhao et al., 2014). Recent studies have underscored the feasibility of co-cultivation techniques for biodiesel production at lower costs. For example, a co-culture of *Chlorella* sp. and *Monoraphidium* sp.*,* showed significant increases in total biomass productivity (62 mg.L^-1^. d^-1^), total lipid content (47.72%), and lipid productivity (29.52 mg.L^-1^. d^-1^) compared to monoculture systems (Zhao et al., 2014). An overview of different methods employed for microalgae biomass, lipid production, and wastewater treatment is provided in Table 4.

**TABLE 4:** Microalgae-microalgae co-culture systems used for different applications.

Microalgae/microalgae	Product/application	Culture method	Yield	Reference
*Scenedesmus* sp. 336/*C*. *sorokiniana*	Brewery wastewater treatment	250 mL conical flask, 6,000 lx, and the light cycle of 24:0	NH3-N, TN, total protein, and COD removal up to 96.22%, 90.57%, 97.37% and 78.83%, respectively	Han et al. (2021)
*C. vulgaris*/*S*. *dimorphus*	Biomass production, lipid producion and Wastewater treatment	1 L photobioreactor, 200–380 μE m^-2^s^-1^ light intensity	0.4 g.L^-1^ biomass concentration, 25.5% lipid content, 70.7% nitrate removal, and 56.8% phosphate removal	Asmare et al. (2014)
*C. vulgaris*/*S*. *obliquus*; and *Chlorococcum* sp	Ethanol production	3 L photobioreactor the light intensity of 4,000 lux for 24 h	58% ethanol production	Juliarnita et al. (2018)
			Carbohydrate production was 12.2 mg.L^-1^	
*Chlorella* sp. HS-2/*Ettlia* sp	Biomass and biodiesel production	1 L bubble column photobioreactor, CO_2_-mixed air (2%)	Biomass productivity 0.7 g.L^-1^.day^-1^, Fatty acids in co-culture was higher than mono-culture of *Ettlia* sp.and lower than the monoculture of *Chlorella* sp. HS^−2^ in mixotrophic condition and was no difference in autotrophic condition	Rashid et al. (2019)

## 6 In-depth analysis of omics studies and consortia: Merging recent trends with a roadmap to microalgae co-cultivation optimization for sustainable applications

Emerging strategies such as photo-bioreactor configuration have been proposed for wastewater treatment, utilizing anoxic/aerobic-algal/bacterial consortia to facilitate the rapid settling of algal/bacterial populations and the removal of nitrogen, organic, and inorganic carbon, with effective biomass recycling (Alcántara et al., 2015). However, the chemical treatments and biomass recovery processes associated with this approach are still expensive. To mitigate these costs, a nutrient remediation and recovery method was suggested that involves a synergistic co-culture of eukaryotic and prokaryotic microorganisms, which maximizes biomass production while minimizing associated expenses (Wicker and Bhatnagar, 2020). Significant progress has been made in omics analysis and metabolic engineering of microalgae and bacterial strains, allowing for the construction (Crozet et al., 2018) and standardization of new pathways in model microbes (Mishra et al., 2019). These methodologies represent an important milestone towards harnessing the potential of photoautotrophic/bacterial co-cultures, particularly with respect to understanding the molecular mechanisms underlying co-cultivation systems and ensuring their stability and productivity.

Research studies that focus on physiological characteristics within consortia have become increasingly important in the context of implementing cell-to-cell communication. These studies utilize advanced techniques such as microscopy, mass spectrometry, quorum sensing, as well as molecular and genetic engineering to gain a better understanding of consortia interactions (Brenner et al., 2008). Genomic approaches are also utilized to identify species composition, genetic variability, and to compare different species within the consortia (Gou et al., 2020). However, these techniques have limitations in that they require specific genomic libraries and are unable to isolate low-abundant species in natural or synthetic consortia. High-throughput proteomic and enzymatic studies have also been employed to improve the understanding of the relationships between genetic and biochemical information, as well as regulatory mechanisms in consortia (VerBerkmoes et al., 2009). While omics approaches are highly sensitive, they are also expensive and require skilled professionals for sample preparation, data analysis, and problem-solving. However, this information is essential for better understanding microbial interactions, optimizing the use of available substrates, increasing productivity, and addressing cultivation optimization problems. Genome-editing approaches offer promising potential for generating more efficient microalgae/bacteria consortia in the future. Currently, microbial consortia systems face challenges in the development and consolidation of computational and mathematical assistance for co-culture realization, which strongly affects the total costs and required time for large-scale productions, a critical criterion for the synthesis and commercialization of bioproducts (Scognamiglio et al., 2021). An open-access database that provides relevant metadata about tested consortia, including descriptions of strains, growth dynamics, biomolecules released, data related to bioprocess conditions in bioreactors, and possible metabolic and omics outcomes, would undoubtedly contribute to improving the current scenario and expanding the applications of microalgae-bacteria consortia in biotechnological applications. In addition, meta-secretomics analysis has proven beneficial in identifying total surface-bound proteins and secretions in consortia. Through the use of reliable and reproducible metabolomics techniques, qualitative and quantitative data can be achieved regarding the metabolites produced by the consortia (Adav et al., 2012).

Moreover, the cultivation of microalgae consortia with bacteria, fungi, or other microalgae relies on the appropriate provision of nutrient, light, and water conditions for optimal biomass yield. By analyzing the intricate dynamics and interactions of these consortia through the lens of omics techniques, we can classify them as either microalgae-assistant or microalgae-dominant. Omic studies influence significantly our understanding of nutrient uptake and CO_2_ assimilation across different microalgae species (Van Den Hende et al., 2012). Some microbial species are able to tolerate high concentrations of supplemented CO_2_, usually ranging from 14 to 100 percent of dissolved gas, while growth inhibition occurs once the supplied CO_2_ concentration exceeds the maximum cellular capacity (Salih, 2011). High CO_2_ tolerant freshwater microalgae strains, in their natural habitats, can survive in a CO_2_-rich environment (up to 30% CO_2_) with better biomass yield and CO_2_ bio-fixation rate, although decreased carotenoid content was reported at the highest CO_2_ level (Swarnalatha et al., 2015). Omic insights therefore can play a pivotal role in the modulation of growth conditions and the improvement of biomass yield.

In the complex microcosm of carbon utilization, microalgae have shown a remarkable capability to metabolize various sources, including alcohol, sucrose, and glucose, in addition to CO_2_ (Tan et al., 2018).

Omics techniques unravel the mechanistic interplay involved in this process, such as the triggering of the Carbon Concentrating Mechanism in aquatic environments. Omics reveal that overexpression of diverse Carbonic anhydrase enzymes is responsible for improving CO_2_ uptake, ultimately favoring the high photosynthetic efficiency of the cells (Kuken et al., 2018). Omics have also provided insights into the effect of high CO_2_ conditions and the modulation of protein secretion in microalgae in flue gas concentrations (>3%) (Baba et al., 2011), indicating the occurrence of vegetative and gametic forms of microalgae when bacteria respiration exceeds the mentioned CO_2_ air level or co-cultivation systems have higher CO_2_ supplements. Insights from omics analysis highlight the importance of sufficient carbón supply in the form of salts (Acién et al., 2017). or CO_2_ rich air for maintaining high productivity in biomass yield (Cho et al., 2011).

The omics techniques further underscore the potential of different organic carbon sources, like non-edible lignocellulosic biomass, agricultural waste, and glycerol from biodiesel production, for cost-effective cultivation. (Patel et al., 2016). Furthermore, omics have revealed how the manipulation of carbon-to-nitrogen (C/N) ratio can induce nitrogen starvation and subsequently increase the lipid content of microalgae (Liu et al., 2008). In a co-culture system of *C*. *pyrenoidosa* and *R. glutinis* in a BBM medium, an increase in C/N ratio from 16 to 64 led to an increase in biomass (from 2.92 to 6.12 g.L^-1^) and lipid content (from 25% to 40.55%) (Liu et al., 2018). The optimal biomass concentration and lipid productivity (90 mg.L^-1^. day^-1^) in the co-culture of *C. pyrenoidosa* and *R. glutinis* was attained at a ratio of 3:1, with total fatty acid (TFA) content twice that of a monoculture. Optimal conditions for the co-cultivation of microalgae and yeast *R. glutinis* have been shown to be at a C/N ratio of 64, resulting in a biomass concentration of 6.12 and a lipid yield of 2.48 (Liu et al., 2018). Omics studies have revealed that nitrogen deprivation can significantly affect the primary and secondary metabolism of microalgal cells, leading to reduced protein synthesis, photosynthetic capacity, and cell growth while increasing the production of neutral lipids (Schmollinger et al., 2014).

Omics technologies have also shed light on the role of stress conditions, such as salt stress, and nitrogen levels in lipids accumulation (Mastrobuoni et al., 2012). The identification and selection of suitable microalgae and fungi strains that can withstand these stress conditions while maintaining productivity are crucial (de Oliveira Magalhães et al., 2022). Therefore, for the co-cultivation of microalgae consortia for synthesizing secondary metabolites, it is essential to ensure a proper nitrogen source supply (>0.35 mM) (Prajapati et al., 2016). For instance, *R*. *glutinis* is known for its ability to produce lipid and β-carotene, while *C*. *tropicalis* is an oleaginous yeast that can grow on various inexpensive agricultural raw materials (Liu et al., 2018). The use of consolidated bioprocessing (CBP), which employs an ethanologenic and a cellulolytic strain for bioethanol production, is another approach to co-cultivation (Park et al., 2012). The co-culture of *C. vulgaris* and *R. glutinis* improved *C. vulgaris* lipid content to 20.8% using 20 g.L^-1^ glucose, while a co-culture of *S*. *obliquus* and *R. glutinis* enhanced the lipid content of *S*. *obliquus* to 24% (Arora et al., 2019). The use of omic techniques in co-cultivation systems comprising *R. glutinis* and *S*. *platensis* has provided valuable insights that has led to increased biomass and lipid accumulation (Magdouli et al., 2016). Selection of an appropriate medium for microalgae co-cultivation is crucial, with deionized water and artificial ocean water being the most commonly used media (Chia et al., 2019). Artificial media offer greater flexibility in terms of nutrient composition and can be optimized for microalgae cultivation. However, the quest for a sustainable culture medium, aided by omics studies, that can provide nutrients to all consortia members while minimizing pollutant production during cultivation continues (Chia et al., 2021).

Omics technologies can provide key insights into how the availability and quality of light sources play a crucial role in achieving optimal biomass production in microalgae co-cultivation, under photoautotrophic and mixotrophic conditions. By understanding the genome, transcriptome, proteome, and metabolome of the microbes involved, we can understand how degree of light penetration is a major factor that influences the efficiency and quality of the consortium (González-Fernández et al., 2011). Additionally, omics allow us to study and understand the significant impact on microalgae-bacteria/fungi co-cultures grown under distinct conditions, such as duration, intensity, and potential limitations of light. As microalgal cells become more densely packed, they can cast shadows on each other and impede light penetration, creating light-limited conditions. In such situations, microalgae can thrive in mixotrophic or heterotrophic modes, depending on the presence of organic carbon sources. Therefore, supplementing organic carbon sources, such as acetate or sugars, instead of improving the light source, is a critical alternative for such growth (Tandon and Jin, 2017). Furthermore, light intensity can influence the interaction between microalgae and bacteria or fungi. Higher light intensity has been found to enhance microalgal growth and thus promote increased biomass yield (Zhai et al., 2017). On the other hand, increased light intensity favored microalgae growth while limiting the nitrification process in the microalgae-bacteria culture. In contrast, a non-graduated temperature increase (up to 32°C) under light intensities (up to 55 µE) promoted the proliferation of the nitrifying bacteria and nitrite and nitrate accumulation (Gonzalez-Camejo et al., 2018). The optimization of light supply is a critical factor in the growth and productivity of photosynthetic microorganisms. While light is the primary energy source for photosynthesis, excessive light levels, coupled with inappropriate temperature or high oxygen concentrations, can negatively impact the photosynthesis process, leading to reduced growth rates (Fernández et al., 2001). Most microalgae perform photosynthesis at 100 to 500 µE, but optimal productivity is achieved at constant irradiance levels in the range of 50–100 µE (Vejrazka et al., 2011). Saturation points are reached at irradiance levels higher than 300 µE in several strains, resulting in photoinhibition (Acién et al., 2017). The impact of light quality and intensity on microalgae metabolism has been studied experimentally and through modeling, demonstrating that even moderate light intensities can alter the set of metabolites produced by cells, leading to reduced availability of nuclear transcripts, proteins, and metabolites, with little to no change in plastid transcripts (Mettler et al., 2014). These findings suggest that microalgae products synthesized at the plastids may be resistant to fluctuations in light intensity during co-cultivation.

Microalgae growth is not only dependent on light intensity but also on wavelength and photoperiod (Iasimone et al., 2018). As light is essential for metabolic activity under photo and mixotrophic conditions, this culture parameter significantly affects their growth (Schulze et al., 2014). The effect of light on cell growth metabolism varies among different species or strains. In a microalgae consortium composed of *C. vulgaris*, *P. subcapitata*, *M*. *aeruginosa*, and *Synechocystis salina*, the optimum daily irradiance of light on nutrients uptake and growth was found to be 208 µE (Gonçalves et al., 2016). Therefore, in general terms, light irradiance levels should be maintained between 50 and 200 µE to avoid photoinhibition. However, the behavior of microalgae under different light conditions and the optimal light intensity and irradiance can be selected based on models of cell growth simulations under different light sources. Additionally, the possible type of metabolites produced by the strains under specific light conditions provided to the consortia can be observed and coordinated with the simulation models (Chang et al., 2011).

The supply of essential nutrients like nitrogen, phosphorus, and trace metals is critical for the growth of both microalgae and bacteria in a consortium (Markou et al., 2014). The bacteria present in the consortia are known to facilitate the breakdown of various nitrogen-containing compounds, which aid in the proliferation of microalgae (Zhou et al., 2013). For instance, the co-cultivation of the bacteria *Azotobacter vinelandii* with microalgae strains *Neochloris* sp. and *Scenedesmus* sp. has been found to result in a commensal relationship where the bacteria provide nitrogen for microalgae growth (Delgadillo-Mirquez et al., 2016).

Omics studies illustrate how nitrogen forms and trace metals play a crucial role in physiological processes like anti-oxidative defense and nutrient uptake (Oliveira et al., 2020). The presence and concentration of nitrogen and phosphorus in the culture medium are critical as they are fundamental building blocks for enzymes and nucleic acid synthesis (Arora et al., 2019). The nutrient constraint has a significant impact on the chemical compositions of microalgae. An optimal growing medium for microalgae must contain carbon (C), nitrogen (N), phosphorus (P), and iron (Fe), although this may vary depending on the specific species. Carbon is an essential component of all biomolecules synthesized by microalgae, including carbohydrates, proteins, nucleic acids, vitamins, and lipids, and is thus necessary in high concentrations (Richmond, 2008). Certain species of mixotrophic microalgae can also use organic compounds such as sugars, acids, and alcohols as carbon sources. Nitrogen plays a vital role in the formation of structural and functional proteins and is a significant component in the production of proteins, nucleic acids, vitamins, and photosynthetic pigments (Richmond, 2008). Nitrogen, a crucial nutrient for microalgae growth, can be supplied in various forms, including inorganic forms such as NO_3_, NO_2_, NO, NH_4_^+^, or organically via urea or amino acids (Perez-Garcia et al., 2011). Among the nitrogen sources, ammonium is the preferred nitrogen source for two microalgae species, *C. vulgaris* and *Pseudokirchneriella subcapitata,* due to its easier assimilation and less energy requirement (Jia and Yuan, 2016). When microalgae are nitrogen-limited, it can affect the synthesis of antioxidants, as found in *Phaeodactylum tricornutum*, *Tetraselmis suecica*, and *C. vulgaris*, where the content of chlorophyll a in biomass was lower under nitrogen-limited condition (Goiris et al., 2015). Along with macronutrients, microalgae growth medium should contain essential micronutrients such as Mg, S, Na, Cl, Ca, Fe, Mo, Mn, Zn, Cu, B, and Co, with an emphasis on magnesium (Mg), sulphur (S), and iron (Fe) (Markou and Georgakakis, 2011).

Fungi and microalgae exhibit opposite zeta potentials, which is the charge developed at the interface of a solid and liquid medium. When the pH is lower, the zeta potential of the co-culture system tends towards neutrality, facilitating electrostatic neutralization and fast fungal spore aggregation. Therefore, slightly acidic environments have shown to be more effective for bio-flocculation (Zhang and Zhang, 2016). However, the efficiency of microalgae-fungi flocculation depends on several other factors, including the carbon source, light, and the combination of microorganisms in the culture (Gultom et al., 2014). The optimal pH selection should be based on the specific applications and harvesting methods since different species exhibit varied metabolic responses to pH changes, leading to varying flocculation efficiency (Prajapati et al., 2016). While some studies have reported that alkaline conditions were more suitable for flocculation than neutral or acidic ones (Leng et al., 2021), others found little effect of pH on flocculation efficiency (Li et al., 2017; Pei et al., 2021). For the co-cultivation of microalgae and yeast, a pH of 5 was reported as optimal (Liu et al., 2015; Arora et al., 2019). The composition and buffering capacity of the culture medium, the dissolved CO_2_ amount, temperature, and the metabolic activity of cells can influence the pH of the culture medium (Singh and Dhar, 2011). The pH tolerance of microalgae culture media is species-specific and can significantly impact growth rates (Zhu, 2015). Optimal pH levels for most microalgae cultures fall within the neutral to slightly alkaline range (pH 7.0–10.0), with some species having an optimal pH as low as 3.0 (Lu et al., 2014). However, beyond these pH ranges, the yield of microalgae is significantly reduced. In the absence of carbonate ion, *Dunaliella bardawil* and *Chlorella ellipsoidea* have been shown to grow at pH levels exceeding 10.0 (Khalil et al., 2010). Aside from pH, other factors such as temperature, agitation, and initial inoculum concentration can impact the composition and stability of a microalgae-based consortium.

Temperature constitutes a paramount environmental factor modulating the proliferation rate, cellular dimensions, biochemical composition, and nutritional requisites of microalgae. Microalgae assimilate thermal energy originating from luminous sources, engendering an elevation in the internal temperature milieu of the culture. Consequently, in the context of extensive outdoor cultivation systems, it is crucial to address both photonic irradiance and the concomitant thermal factors (Deb et al., 2017). The optimal thermal range for microalgal cultivation lies between 20 and 35°C, with particular mesophilic species displaying tolerance to temperatures approaching 40°C. The microalgae consortia of *P*. *tricormutum*, *Tetraselmis gracilis*, *Chaetoceros* sp., and *Minutocellus polymorphus* exhibited the best growth rates within the temperature range of 11°C–36°C, as reported in various studies (Sigaud and Aidar, 1993). Microalgal cultivation is significantly impacted by seasonal changes, which cause temperature fluctuations during the day/night cycle. Small-scale reactors, which operate in relatively cooler ambient temperatures, do not require temperature control, as the input of heat through radiation is balanced by the output through circulation. However, large-scale outdoor reactors experience high levels of solar radiation, necessitating the use of different heat control devices to maintain the optimal temperature (Huesemann et al., 2013).

When utilizing fungi pellets for flocculating microalgae in microalgae/fungi co-cultivation, it is widely accepted that a higher concentration of fungi (higher inoculation ratio to microalgae) is favorable for efficient harvesting (Liu et al., 2008). However, when fungal spores are directly mixed with a microalgal solution, increasing spore concentration may not always improve flocculation efficiency (Liu et al., 2008). At excessively high spore concentrations, microalgae-fungal pellets cannot form, leading to low harvesting rates. The initial inoculum concentration of bacteria/fungi/microalgae plays a critical role in the formation of fungi-microalgae pellets and the growth of microalgae-bacteria, which further affects biomass yield and metabolite production (Yang et al., 2019). This may be due to the interaction between fungal hyphae during the early growth stages, which prevents pellet formation under high initial inoculum concentrations (Zhao Y. et al., 2019). Additionally, the size of the fungal spore inoculation is linked to the pH of the medium, which indirectly influences flocculation efficiency (Zhao et al., 2013). In the co-cultivation of *Chlorella* sp. and *S. cerevisiae*, the effect of different inoculum ratios of microalgae on yeast (1:2, 1:1, 2:1) has a significant impact on the final biomass, oil production, and specific production yield (Y_p/x_). The best results were obtained at a ratio of 2:1 for all studied parameters, but with only a slight increase compared to the 1:1 inoculum ratio (Shu et al., 2013). In the co-culture of *C. pyrenoidosa* and *R. glutinis* in piggery wastewater, the optimal ratio of yeast to algae was found to be 3:1, resulting in 36.07% TN (total nitrogen) removal, 58.53% ammonium nitrogen removal, 33.20% TP (total phosphate) removal, and 56.25% COD (chemical oxygen demand) removal after 6 days (Li et al., 2019). The highest biomass concentration and photosynthesis activity for co-cultivating *S*. *obliquus* and *C*. *Tropicalis* were achieved at a microalga to yeast ratio of 3:1 (Wang R. et al., 2016).

Proper agitation is a critical factor in the morphology of fungi, which plays a significant role in the harvesting efficiency of the co-culture system (Porcel et al., 2005). Certain studies have reported that low agitation speed facilitates the attachment of microalgae to fungi, overcoming electrostatic repulsion (Choi, 2015; Bhattacharya et al., 2017). The co-cultivation of *C. vulgaris* and *R. glutinis* reported 150 rpm as the optimum agitating speed, with further increase having no significant effect on biomass and lipid production (Cheirsilp et al., 2011). Photobioreactor design is another crucial parameter to consider in a co-culture system. Higher biomass density than monocultures greatly influences mass transfer rates, thereby impacting growth. Designing a cultivation system with improved mass transfer efficiency, ease of scale-up, and lower costs is thus essential, with nanoparticles offering a promising solution to this end (Magdouli et al., 2016).

We therefore argue that the use of omic technologies in the optimization of microalgae co-cultivation can provide critical insights that can significantly enhance sustainable applications. Omics provide valuable insights into the influence of various factors such as carbon and nitrogen sources, light conditions, and temperature on the microalgae consortia’s performance. It helps unravel the metabolic interactions within the consortia and identify the key parameters for successful co-cultivation. Thus, omics research plays a pivotal role in improving microalgae co-cultivation for sustainable applications. More extensive use of these techniques in conjunction with experimentation will allow for the refinement of the cultivation conditions and contribute to the optimization of microalgae co-cultivation, ultimately contributing to the attainment of sustainability goals.

## 7 Conclusion

Co-cultivation systems incorporating microalgae have risen to prominence as a resource platform for an array of biotechnological applications, which span the spectrum from biodiesel production to wastewater treatment. These systems have a remarkable ability to thrive on inexpensive feedstocks such as cellulosic biomass, thereby reducing operational costs substantially. Yet, to translate microalgae cultivation into a large-scale operation, we need a more profound understanding of cultivation methods, metabolite recovery and purification from co-culture systems, and strategies to bolster culture resilience. Resilience, in this context, refers to the ability of the co-culture system to withstand changes in environmental conditions and recover from disturbances. The resilience of co-cultivation systems is essential for sustainable large-scale operations.

Future research should focus on optimizing the selection of cost-effective feedstock and harvesting methods, and on the evaluation of varying operating conditions. These include factors such as pH, temperature, light intensity, nutrients, and carbon availability that govern the successful operation of these systems. These steps will be crucial to achieve the economic feasibility of large-scale production of high-value microalgae products. While the significance of microalgae cannot be understated, it is equally important to acknowledge the critical role played by the associated organisms in the co-culture system. The success of these co-cultivation systems hinges not only on the algal components but also on the symbiotic relationship established with other microbial members. Their contributions are fundamental in enhancing the productivity and overall functionality of these systems.

Advancements in the field of omics have opened up new horizons for microalgae cell analysis and present promising prospects for co-cultivation systems. Continuous research efforts in this domain can potentially unveil intricate details about the interactions within these co-cultures, contributing substantially to our comprehensive understanding of these systems. To conclude, the sustainable synthesis of diverse products at an industrial scale lies in the successful implementation of microalgae co-cultivation. This vision, however, can only be realized through an in-depth understanding of the co-culture system as a whole, focused optimization of operating conditions, and leveraging the advancements in omics technology.
